# Cumulative live birth following preimplantation genetic testing for aneuploidy versus conventional IVF/ICSI in unexplained recurrent pregnancy loss: a retrospective cohort study with intention-to-treat and per-protocol analyses

**DOI:** 10.3389/fendo.2026.1910686

**Published:** 2026-09-30

**Authors:** Dan Han, Zuying Xu, Wenqiang Shen, Ye He, Haoyu Chen, Huifen Xiang, Ping Zhou, Yunxia Cao

**Affiliations:** 1 Department of Obstetrics and Gynecology, NHC Key Laboratory of Study on Abnormal Gametes and Reproductive Tract, the First Affiliated Hospital of Anhui Medical University, Hefei, China; 2 Engineering Research Center of Biopreservation and Artificial Organs, Ministry of Education, Hefei, China; 3 Key Laboratory of Population Health Across Life Cycle (Anhui Medical University), Ministry of Education of the People’s Republic of China, Hefei, China; 4 Anhui Provincial Key Laboratory of Reproductive Disorders and Obstetrics and Gynaecology Diseases, Hefei, China; 5 The Second Clinical Medical College, Anhui Medical University, Hefei, China

**Keywords:** cumulative live birth rate, intention-to-treat analysis, inverse probability of treatment weighting, mosaic embryo, preimplantation genetic testing for aneuploidy, recurrent pregnancy loss

## Abstract

**Objective:**

To compare cumulative live birth rates (CLBR) between preimplantation genetic testing for aneuploidy (PGT-A) and conventional IVF/ICSI in women with unexplained recurrent pregnancy loss (uRPL) using intention-to-treat (ITT) and per-protocol (PP) analyses, and to assess effect modification by maternal age and prior miscarriage number.

**Methods:**

This single-center retrospective cohort (2018–2024) included women with uRPL undergoing their first oocyte retrieval with planned single-blastocyst transfer. The ITT population included 589 patients (342 PGT-A vs 247 IVF/ICSI); after excluding patients who never underwent transfer and 47 patients (16 PGT-A, 31 IVF/ICSI) with remaining cryopreserved embryos and no live birth at the follow-up cutoff, the PP population included 389 patients (221 vs 168) who completed at least one transfer. Stabilized inverse probability of treatment weighting with doubly robust adjustment and robust variance estimation was applied. The primary outcome was CLBR per oocyte retrieval.

**Results:**

In the ITT population, unadjusted CLBR was comparable (48.5% vs 47.4%, P = 0.779) and remained non-significant after adjustment (aOR 0.862, 95% CI 0.591–1.255, P = 0.437). In the PP population, neither first-transfer live birth (63.8% vs 58.3%, P = 0.272; aOR 0.942, P = 0.814) nor CLBR (75.1% vs 69.6%, P = 0.230; aOR 0.858, P = 0.595) differed significantly. Age and prior miscarriage number did not significantly modify the treatment–CLBR association (age: ITT P = 0.871, PP P = 0.071; miscarriage number: ITT P = 0.695, PP P = 0.289; both miscarriage-number tests were exploratory owing to limited propensity-score overlap in the >2-miscarriage stratum). Among women aged ≥35 years, ITT CLBR did not differ (33.6% vs 29.7%, P = 0.534; aOR 0.846, 95% CI 0.447–1.603, P = 0.608). Unadjusted PP outcomes favored PGT-A among women aged ≥35 years and those with exactly two prior miscarriages, but no adjusted differences remained significant after Bonferroni correction, except first-transfer clinical pregnancy in the ≥35-year stratum, which requires cautious interpretation. Miscarriage rates did not differ significantly between groups; no statistically significant perinatal differences were observed in this sample (166 vs 117 deliveries).

**Conclusions:**

PGT-A did not improve CLBR per oocyte retrieval in women with uRPL and should not be routinely recommended. Unadjusted PP subgroup advantages are more plausibly attributable to embryo attrition and residual confounding than true benefit and require prospective confirmation.

## Introduction

*In vitro* fertilization–embryo transfer (IVF–ET) and intracytoplasmic sperm injection (ICSI) are cornerstone treatments for infertility. Recurrent pregnancy loss (RPL), defined by the European Society of Human Reproduction and Embryology (ESHRE) as the loss of two or more pregnancies, affects approximately 1–2% of reproductive-age couples and remains a major challenge in the management of assisted reproductive technology (ART) (1, 2). The etiology of RPL is multifactorial, including genetic abnormalities, uterine anomalies, endocrine disorders, immune dysfunction, thrombophilia, and infections. However, in approximately 50% of cases, no identifiable cause is found, resulting in a diagnosis of unexplained recurrent pregnancy loss (uRPL) (3).

Preimplantation genetic testing for aneuploidy (PGT-A) is a screening technique that involves trophectoderm biopsy of blastocysts, typically performed on day 5 or 6 of embryonic development. By assessing chromosomal status, PGT-A enables the selection of euploid embryos for single embryo transfer with the goal of increasing live birth rate per transfer while reducing the risks of miscarriage and multiple gestation (4). Some studies also indicate that PGT-A could enhance cumulative live birth rates and reduce the number of transfer cycles required per live birth (5, 6). Nevertheless, reported outcomes for PGT-A vary widely across studies (7). Evidence from a large randomized trial in the general infertile population (8) and observational studies in women with RPL (9, 10) has shown comparable cumulative live birth rates (CLBR) between PGT-A and conventional IVF/ICSI, questioning its universal benefit. Moreover, PGT-A adds procedural steps that can delay time to live birth without improving overall reproductive success (9).

Emerging evidence indicates that the efficacy of PGT-A varies considerably across patient subgroups. Advanced maternal age (AMA) is associated with an exponential rise in embryonic aneuploidy rates (11), which may enhance the benefit of chromosomal screening in older women. In contrast, the relationship between prior miscarriage history and embryonic ploidy remains controversial: young patients with idiopathic RPL exhibit higher blastocyst aneuploidy rates than controls (12), whereas the number of prior pregnancy losses does not appear to influence euploidy rates (13). In addition, PGT-A is subject to inherent treatment attrition: a proportion of patients fail to develop biopsiable blastocysts or have no euploid embryo available, and therefore never reach embryo transfer (14, 15). Analyses restricted to patients who actually undergo transfer (i.e., per-protocol or per-transfer analyses) may consequently overestimate the benefit of PGT-A, whereas intention-to-treat analyses anchored at treatment initiation provide a less biased estimate of its clinical utility (14). However, studies that apply both analytical frameworks while rigorously controlling for confounding by indication remain scarce. Current literature on PGT-A in RPL is further limited by heterogeneous study populations and a predominant focus on per-transfer outcomes rather than CLBR per oocyte retrieval (16, 17). Indeed, improvements in per-transfer live birth rates do not necessarily translate into higher CLBR per oocyte retrieval (18). Such a narrow focus may overlook patients who never reach embryo transfer or who require multiple cycles to achieve a live birth. Furthermore, comprehensive subgroup analyses stratified by maternal age and number of prior miscarriages—two key potential effect modifiers—remain limited, and the safety profile of PGT-A with respect to perinatal outcomes in women with uRPL has rarely been reported (16, 18, 19).

Therefore, this retrospective cohort study aimed to compare reproductive and perinatal outcomes between PGT-A and conventional IVF/ICSI in a well-characterized, homogeneous population of women with uRPL, using both intention-to-treat and per-protocol analytical frameworks with inverse probability of treatment weighting to minimize confounding by indication. We further sought to determine whether the effect of PGT-A on CLBR and first-transfer outcomes is modified by maternal age and the number of prior miscarriages, in order to identify specific subgroups that may derive differential benefit or harm from chromosomal screening.

## Materials and methods

### Study design and setting

This study included patients with uRPL who received fertility treatment at the Reproductive Medicine Center of the First Affiliated Hospital of Anhui Medical University between January 2018 and December 2024. All enrolled patients underwent their first oocyte retrieval. The choice of treatment protocol (PGT-A or conventional IVF/ICSI) was made by patients themselves after comprehensive counseling by a certified genetic counselor, which covered the principles, potential benefits, limitations, and potential risks of both approaches. Based on this decision, patients were assigned to one of two groups: in the PGT-A group, euploid blastocysts were prioritized for transfer; if no euploid embryo was available, a blastocyst with low-level mosaicism could be transferred after obtaining thorough informed consent. In the IVF/ICSI group, blastocysts with the highest morphological scores were preferentially selected for transfer. The follow-up for frozen embryo transfer was censored on May 31, 2026. This report adheres to the STROBE recommendations for cohort studies (20), and a completed STROBE checklist is provided as **Supplementary Material**.

### Study population and eligibility

The study applied stringent selection criteria. Patients were included only if they met all of the following conditions: (1) a diagnosis of RPL, defined as the loss of two or more pregnancies, excluding ectopic pregnancy and molar pregnancy; (2) initiation of their first oocyte retrieval cycle during the study period; and (3) a planned single-blastocyst transfer strategy, with no more than one blastocyst transferred per cycle.

Exclusion criteria were as follows: (1) use of donor sperm or oocytes; (2) known chromosomal abnormalities (e.g., balanced translocations or inversions); (3) anatomical anomalies of the female reproductive tract, uterine fibroids, or adenomyosis; (4) endocrine disorders, autoimmune diseases, or hematologic disorders; (5) history of smoking, alcohol consumption, or documented exposure to toxic substances; (6) cleavage-stage embryo transfer or double blastocyst transfer. Applying these criteria yielded a clearly defined and homogeneous study cohort (**Figure 1**).

**Figure 1:**
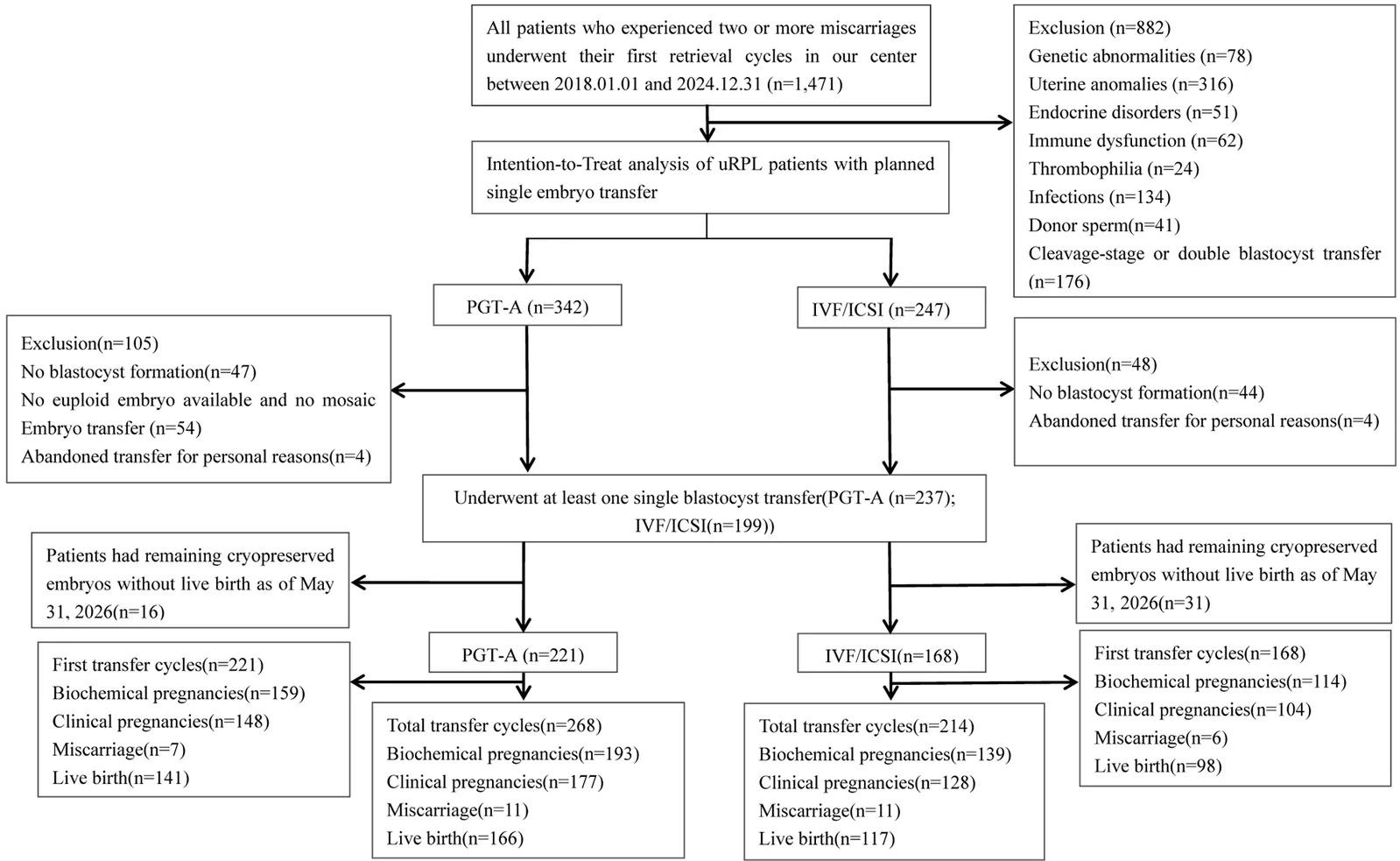
Flowchart of patient enrollment, treatment allocation, and reproductive outcomes in the ITT and PP populations. Of the 62 patients without euploid embryos, 8 underwent mosaic embryo transfer and 54 did not proceed to transfer.

After screening, the intention-to-treat (ITT) population comprised 589 patients who initiated their first oocyte retrieval cycle and were scheduled for single blastocyst transfer: 342 in the PGT-A group and 247 in the conventional IVF/ICSI group. In the PGT-A group, 47 patients had no blastocyst formation, 54 had no euploid embryos available and did not undergo mosaic embryo transfer, 4 abandoned transfer for personal reasons, and 16 still had cryopreserved embryos without having achieved a live birth as of the follow-up cutoff on May 31, 2026; in the IVF/ICSI group, 44 had no blastocyst formation, 4 abandoned transfer, and 31 still had cryopreserved embryos without having achieved a live birth as of the same cutoff. These patients were retained in the ITT analysis: those who never proceeded to embryo transfer were considered as not having achieved a live birth, whereas those with remaining cryopreserved embryos were treated as not having achieved a live birth at the follow-up cutoff for the calculation of the conservative cumulative live birth rate, and as having achieved a live birth for the calculation of the optimistic cumulative live birth rate. The per-protocol (PP) population comprised patients who completed at least one single blastocyst transfer, excluding those who still had cryopreserved embryos without having achieved a live birth—yielding 221 cases in the PGT-A group and 168 cases in the IVF/ICSI group—and was used to evaluate the biological efficacy of the two techniques.

This study was approved by the Ethics Committee of the First Affiliated Hospital of Anhui Medical University (Approval No. 20211217).

### PGT-A and IVF/ICSI treatment

Ovarian stimulation, oocyte retrieval, fertilization, embryo culture, blastocyst morphological assessment, trophectoderm (TE) biopsy, embryo vitrification and thawing, frozen–thawed embryo transfer, and luteal phase support were all performed as previously described by researchers at our center (21, 22). The Gardner–Schoolcraft grading system was used to assess blastocyst quality, and blastocysts scoring ≥3BB were defined as good-quality blastocysts (23). Blastocysts with a Gardner score of ≥4BC on day 5 (D5) or day 6 (D6) were routinely selected for trophectoderm biopsy. When no D5 or D6 blastocysts were available, day 7 (D7) blastocysts scoring ≥3BB were also biopsied. During biopsy, 5–10 TE cells were collected and subjected to whole-genome amplification (WGA) using either the multiple displacement amplification (MDA) kit from Qiagen or the SurePlex amplification system from Illumina. The amplified products were sequenced on the Ion PGM and Illumina NextSeq550 platforms.

Bioinformatic analyses were conducted strictly in compliance with the consensus guidelines issued by the European Society of Human Reproduction and Embryology (ESHRE) PGT Consortium (24). Briefly, the genome was divided into non-overlapping windows ranging from 600 kb to 1 Mb, and reference baselines were established using sequencing data from euploid embryos to calculate copy number ratios; copy number variations (CNVs) ≥4 Mb were defined as definitive chromosomal abnormalities. Embryos were stratified by mosaic proportion: embryos with a mosaic ratio of 20%–80% were classified as mosaic embryos, those with a mosaic ratio <20% as euploid embryos, and those with a mosaic ratio >80% as full aneuploid embryos. All full aneuploid embryos were further categorized into three subtypes: whole-chromosome aneuploidy (monosomy, trisomy, haploidy/polyploidy), segmental aneuploidy (segmental deletion or duplication ≥4 Mb), and complex aneuploidy harboring two or more independent chromosomal aberrations. Mosaic embryos were subdivided into low-level mosaicism (mosaic ratio <50%) and high-level mosaicism (mosaic ratio ≥50%). The <50% threshold for low-level mosaicism was adopted in accordance with the embryo-ranking framework of Viotti et al (25), under which embryos with lower mosaic proportions are prioritized for transfer, and is supported by prospective non-selection data indicating that low- and intermediate-level mosaic embryos retain developmental potential comparable to euploid embryos (26). Of note, the institutional <50% cut-off for low-level mosaicism is broader than the 20%–40% mosaic range to which the PGDIS position statement assigns the most favorable transfer priority (27); the 45%-mosaic embryo transferred in this cohort therefore exceeded the PGDIS priority band but met the institutional criterion for low-level mosaicism, and its transfer followed full genetic counseling and written informed consent, with prenatal diagnosis recommended in accordance with PGDIS guidance (27). Euploid blastocysts were prioritized for frozen-thawed embryo transfer following genetic counseling. For patients without any euploid blastocysts available, low-level mosaic blastocysts could be transferred after the associated risks, including miscarriage and fetal chromosomal anomalies, had been fully disclosed and written informed consent was obtained. In exceptional cases where transfers of euploid embryos had not resulted in a live birth, transfer of a low-level mosaic embryo could also be considered after thorough genetic counseling and written informed consent. High-level mosaic embryos and all subtypes of full aneuploid embryos were not recommended for routine transfer.

### Data collection

Relevant clinical data were extracted from the hospital’s electronic medical record (EMR) system and included: (1) patient baseline characteristics, including age, body mass index, duration of infertility, number of previous miscarriages, antral follicle count, basal FSH, E2 and LH levels; (2) cycle baseline characteristics, such as endometrial thickness on the day of embryo transfer; (3) ovarian stimulation and embryo-related parameters, including total dosage of gonadotropins, the duration of ovarian stimulation, the number of oocytes retrieved, mature oocytes, 2PN zygotes, 2PN cleaved embryos, transferable blastocysts, high-quality blastocysts, developmental day of transferred embryo, and morphological grade of the transferred blastocyst; (4) pregnancy outcome indicators, including biochemical pregnancy, clinical pregnancy, intrauterine pregnancy, miscarriage and delivery status; (5) neonatal characteristics, such as Apgar scores and birth weight; and (6) other important data, including gestational age and pregnancy complications. No missing data were observed for the main variables used in this study.

### Outcome measures

The primary outcome was CLBR. In accordance with the ICMART/WHO glossary and the IMPRINT consensus (28, 29), CLBR was defined as the proportion of patients who achieved at least one live birth from a single oocyte retrieval cycle, including the fresh transfer and all subsequent frozen-thawed embryo transfers, until a live birth occurred or all embryos from that cycle had been used, whichever occurred first. CLBR was calculated as the number of women who delivered a live infant at or after 28 weeks’ gestation divided by the total number of women in either the PGT-A or the IVF/ICSI group. Secondary outcomes included biochemical pregnancy rate, clinical pregnancy rate, miscarriage rate, live birth rate per transfer, and perinatal outcomes. Biochemical pregnancy was defined as a positive serum human chorionic gonadotropin (hCG) test two weeks after embryo transfer. Clinical pregnancy was defined as a gestational sac confirmed by transvaginal ultrasound (3–5 weeks post-transfer) with prior positive and sustained serum β-HCG. In line with the Chinese national definition of live birth, which conforms to the WHO criteria recommended for international comparison (30, 31), fetal loss occurring before 28 weeks’ gestation was classified as miscarriage, and live birth was defined as the delivery of a live infant at or after 28 weeks’ gestation (or birth weight ≥1000 g when gestational age was unknown) following embryo transfer.

### Perinatal outcomes

Trained research nurses conducted regular follow-ups with all patients to determine pregnancy outcomes. Maternal data included detailed documentation of gestational complications, such as gestational hypertension, gestational diabetes mellitus, gestational thyroid dysfunction, premature rupture of membranes, and placenta previa. Delivery details covered the mode of delivery and the exact gestational age at birth. Neonatal outcomes were assessed and included the following indicators: preterm birth status (categorized as preterm [28 to <37 weeks] and very preterm [28 to <32 weeks]), birth weight in grams (with classifications for low birth weight [<2,500 g] and very low birth weight [<1,500 g]), presence of congenital anomalies, and Apgar scores at 1 minute after birth.

### Data analysis

The study data were derived from the electronic medical record system. Statistical analyses were performed separately on two analysis sets: the intention-to-treat (ITT) population and the per-protocol (PP) population (i.e., the transferred population), aiming to elucidate the clinical effects of PGT-A from different perspectives. The ITT analysis aimed to quantify the association of initial treatment allocation with reproductive outcomes from a real-world clinical decision-making perspective, while the per-protocol (PP) analysis only evaluated the relationship between PGT-A and pregnancy outcomes in patients who completed embryo transfer.

For the above research objectives, we adopted different covariate control strategies. In the ITT analysis, cycle-level variables (e.g., endometrial thickness on the day of transfer) and post-treatment variables (e.g., number of oocytes retrieved) were not included in the primary adjustment models to avoid overadjustment bias; one sequential sensitivity specification that additionally adjusts for the number of oocytes retrieved is shown in **Supplementary Table 4** (specification f) and is explicitly subject to overadjustment toward the null or beyond. Given that the first-cycle live birth rate and per-cycle live birth rate are only applicable to patients who have undergone a transfer cycle, whereas the ITT population includes individuals who may not have undergone transfer after initiation of the first oocyte retrieval cycle, this study only assessed the cumulative live birth rate in the ITT population and did not conduct analyses of first-cycle or per-cycle live birth rates. For pregnancy outcome analyses in the PP population, the first transfer cycle served as the primary analysis endpoint, with all transfer cycles additionally included as a sensitivity analysis to assess the robustness of the results. In the PP population analysis, post-transfer mediators—including blastocyst morphological grade, day of embryo transfer, and cycle-level mediators such as endometrial thickness on the day of transfer—were not included in the multivariable adjustment models. Only pre-treatment patient-level variables and post-treatment baseline variables were adjusted, in order to avoid attenuating the estimated association by adjusting for variables on the causal pathway. In the PP population, the number of oocytes retrieved was included as an adjustment variable because it reflects the patient’s baseline ovarian reserve and responsiveness to stimulation—a prognostic factor that is determined before embryo transfer, even though it is measured after treatment allocation. By contrast, endometrial thickness on the day of transfer was not adjusted for, as it represents a direct consequence of the transfer strategy (e.g., fresh versus frozen–thawed cycles) and thus lies on the causal pathway from treatment assignment to the outcome. Adjusting for it would remove part of the treatment effect and bias the estimate toward the null. **Supplementary Table 4** illustrates the overadjustment bias introduced when the number of oocytes retrieved—a post-allocation variable on the causal pathway—is entered into the ITT model; accordingly, this variable was not used for adjustment in the ITT framework.

### Statistical analysis

Statistical analyses were performed using SPSS version 25.0 (IBM Corp., Armonk, NY, USA) and R version 4.5.2 (R Foundation for Statistical Computing, Vienna, Austria) (32). For binary outcomes with repeated measurements, generalized estimating equations (GEE) were fitted using the geeglm function from the geepack package (version 1.3.13) (33, 34), specifying a binomial(“logit”) family, an independence working correlation matrix, and robust sandwich standard errors (empirical variance estimation). For cross-sectional weighted analyses, design objects were constructed using svydesign from the survey package (version 4.5) (35), and weighted logistic regression was fitted using svyglm with a quasibinomial family (to avoid warnings triggered by non-integer weights; point estimates are identical to those from binomial), with standard errors obtained via Taylor linearization (vcov). All reported confidence intervals and P-values are based on the aforementioned robust/design-based variances, rather than model-based standard errors. Continuous variables are presented as median with interquartile range or mean ± standard deviation and were compared using the Mann–Whitney *U* test or independent-samples *t* test, as appropriate. Categorical variables are summarized as counts and percentages and were compared using the chi-square test or Fisher’s exact test. A two-tailed p-value < 0.05 was considered statistically significant.

For variable selection, to avoid the impact of multicollinearity on model stability, we selectively included variables from highly correlated combinations (e.g., number of oocytes retrieved, number of MII oocytes, number of 2PN zygotes, etc.) based on clinical rationality. Propensity scores for treatment assignment were estimated separately for the ITT and PP populations using multivariable logistic regression. Subgroup analyses (by age and number of miscarriages) were performed by including interaction terms between treatment group and subgroup variables in the full-data models, to evaluate the potential modifying effect of these subgroups on the treatment association. Each weighting model incorporated variables with P < 0.05 in univariate comparisons together with covariates considered clinically important, and stabilized inverse probability of treatment weights were applied. Weight truncation thresholds were set at the 1st and 99th percentiles to attenuate the influence of extreme values. Covariate balance before and after weighting was assessed using standardized mean differences (SMDs), with an absolute SMD < 0.1 conventionally considered indicative of adequate balance (**Supplementary Table 2**). In both the ITT and PP analyses, covariates with a post-weighting SMD > 0.1 were further adjusted in the corresponding weighted multivariable regression models—restricted to pre-treatment patient-level variables in ITT, and extended to both pre- and post-treatment patient-level variables in PP—to maintain consistent effect estimates in the presence of residual imbalance. The covariate sets included in each IPTW model and each multivariable model are listed below the corresponding Tables.

Propensity scores were estimated using multivariable logistic regression in SPSS, from which stabilized inverse probability of treatment weights were derived. Standardized mean differences (SMDs) were also calculated in SPSS to assess covariate balance before and after weighting. We performed complex-sample analyses using the R package survey, constructing design objects via svydesign(ids = ~ID, weights = ~w), where ID is the unique patient identifier and w is the IPTW weight. Variance estimation was conducted using Taylor linearization. This approach was used to evaluate the biochemical pregnancy rate, clinical pregnancy rate, miscarriage rate, and live birth rate for the first transfer cycle, as well as the cumulative live birth rate (CLBR) across all cycles. For repeated measures data, we fitted generalized estimating equations (GEE) using the geeglm function from the geepack package, specifying clustering by patient_id, setting corstr = “independence” to obtain consistent estimates of marginal parameters, and using std.err = “san.se” for robust standard errors. Interaction terms between age subgroup and treatment group, and between miscarriage number subgroup and treatment group, were included in the models to assess whether these subgroups served as effect modifiers of PGT-A efficacy, and corresponding subgroup-specific odds ratios and P-values were derived from the same models. Because the number of prior miscarriages nearly determined treatment allocation in this cohort, propensity-score overlap in the >2-miscarriage stratum was limited and the positivity assumption was nearly violated; since an interaction test is a contrast between stratum-specific coefficients, instability of either coefficient propagates to the test. All miscarriage strata × treatment interaction tests were therefore treated as exploratory and should not be interpreted as evidence of effect heterogeneity. To control for multiple comparisons in subgroup analyses, we applied Bonferroni correction separately for two independent effect modifiers—maternal age subgroups and miscarriage number subgroups—based on the definition of a family of tests. Each subgrouping variable was treated as a separate testing family, within which comparisons between the two strata (PGT-A vs IVF/ICSI) were performed. Accordingly, each family contained two tests, and the corrected significance level was set at α’ = 0.05/2 = 0.025 (36). For validation, complex-samples logistic regression results were further examined using SPSS. Given the limited sample size in certain strata (e.g., patients with >2 miscarriages in the PP population), the corresponding effect estimates were interpreted in conjunction with the magnitude of post-weighting SMDs and the width of confidence intervals.

## Results

### Study population

Between January 2018 and December 2024, a total of 1,471 patients with RPL underwent their first cycle of PGT-A or conventional IVF/ICSI treatment at our center. In the intention-to-treat (ITT) population, 589 patients with uRPL met the inclusion criteria, including 342 in the PGT-A group and 247 in the conventional IVF/ICSI group. Ultimately, in the per-protocol (PP) population, 389 patients with uRPL met the inclusion criteria and were included in the analysis: 221 in the PGT-A group and 168 in the conventional IVF/ICSI group (**Figure 1**).

### PGT-A cycle and embryo characteristics

A total of 342 women in the PGT-A group underwent oocyte retrieval cycles. Of these, 47 (13.7%) had no blastocyst formation, while 295 had at least one blastocyst available for biopsy. In total, 1,768 blastocysts were subjected to chromosomal analysis, yielding 585 euploid embryos (33.1%). Among the remaining embryos, monosomy (12.6%), trisomy (11.0%), segmental aneuploidy (11.5%), complex aneuploidy (6.6%), and polyploidy (0.2%) were identified; chromosomal mosaicism was detected in 362 embryos (20.5%), and 82 embryos (4.6%) yielded inconclusive results. Notably, 62 of the 295 patients with biopsied embryos (21%) had no euploid embryo available; of these, 8 subsequently underwent mosaic embryo transfer, while 54 did not proceed to embryo transfer. The detailed distribution of PGT-A results is summarized in **Table 1**. When chromosomal aberrations were enumerated individually—with each affected chromosome contributing one count per embryo—a total of 1,060 chromosome-involvement events were recorded among aneuploid embryos. Chromosome 16 was most frequently involved (8.8%), followed by chromosomes 22 (8.1%) and 21 (7.6%) (**Figure 2**).

**Table 1:** Results of preimplantation genetic testing for aneuploidy (PGT-A) in the PGT-A group of the ITT population.

Result on preimplantation genetic testing	No./total no. (%)
Chromosomal status of biopsied blastocysts (n = 1,768)	
Euploid	585/1,768 (33.1%)
Monosomy	222/1,768 (12.6%)
Trisomy	194/1,768 (11.0%)
Segmental aneuploidy	203/1,768 (11.5%)
Complex aneuploidy	116/1,768 (6.6%)
Polyploidy	4/1,768 (0.2%)
Chromosomal mosaicism	362/1,768 (20.5%)
Inconclusive	82/1,768 (4.6%)
Patients with biopsied blastocysts (n = 295)	
Absence of euploid embryos	62/295 (21.0%)

Categories of chromosomal status are mutually exclusive. Complex aneuploidy refers to abnormalities involving two or more chromosomes. The denominator for absence of euploid embryos is the 295 patients with at least one biopsied blastocyst; all other rows use 1,768 biopsied blastocysts as the denominator.

**Figure 2:**
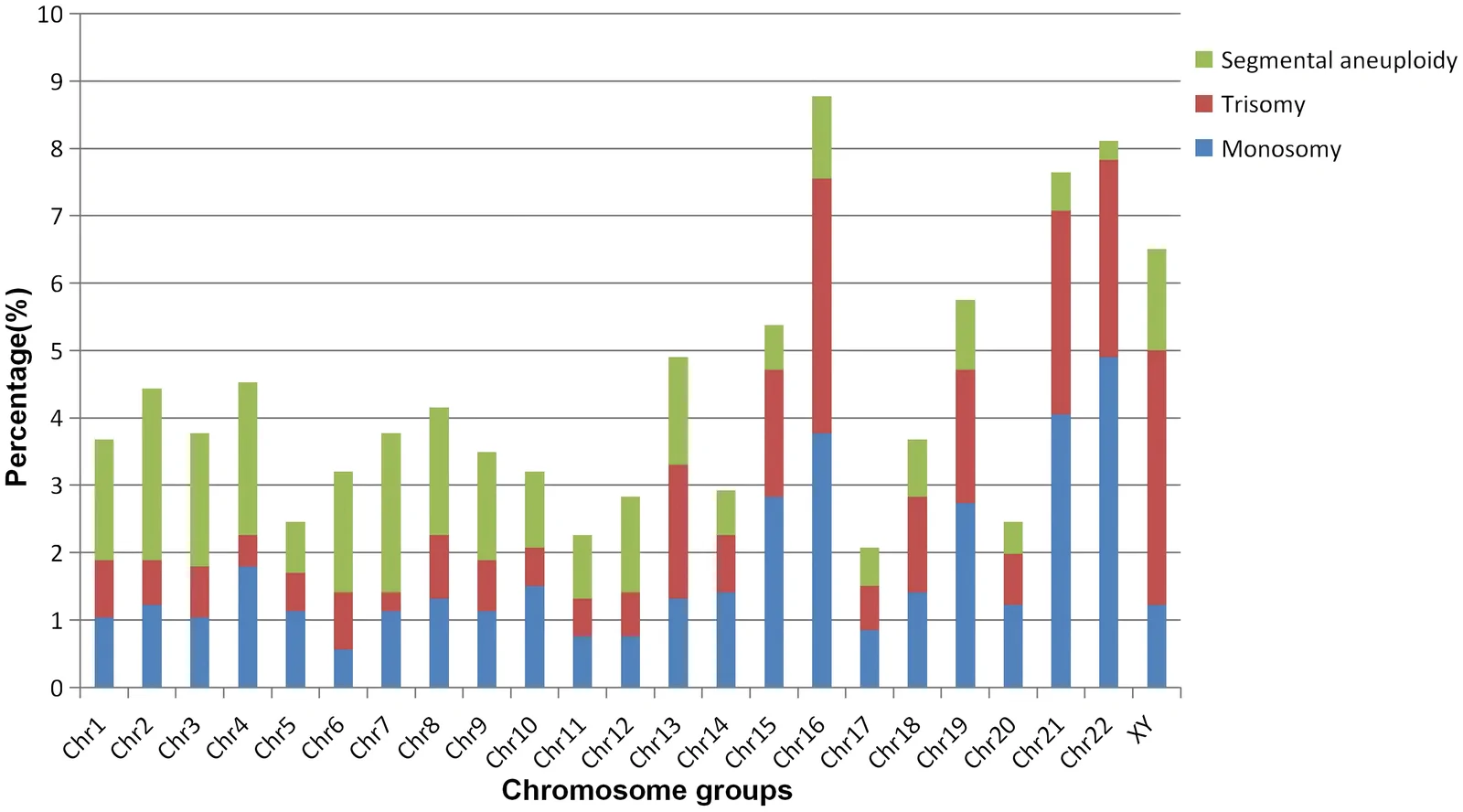
Distribution of involved chromosomes among 1,060 chromosome-involvement events in aneuploid embryos. Percentages are based on 1,060 chromosome-involvement events among aneuploid embryos.

### Outcomes of mosaic embryo transfers in the PGT-A group

In this population, 237 PGT-A patients (the 221 per-protocol patients plus 16 with remaining cryopreserved embryos at the follow-up cutoff) underwent 298 embryo transfer cycles, of which 11 (3.7%) involved the transfer of a mosaic embryo (mosaic level 30%–45%), while the remaining cycles involved euploid embryos. Of these 11 patients, eight had no euploid embryos available, whereas in the remaining three patients the mosaic embryo was transferred in the third transfer cycle after two euploid embryo transfers had not resulted in a live birth. Of the 11 mosaic embryo transfer cycles, seven (63.6%) resulted in clinical pregnancy, one ended in miscarriage, and six (54.5%) resulted in live birth. Among the six live births, four underwent prenatal diagnosis by amniocentesis with karyotyping and chromosomal microarray analysis, all revealing a normal karyotype (46,XN) without pathogenic copy number variations; prenatal diagnosis was not performed in the remaining two cases. All six live births occurred at 36–40 weeks of gestation, with birth weights of 3,050–3,760 g and no low-birth-weight infants observed. Detailed characteristics of the 11 mosaic embryo transfer cycles are summarized in **Supplementary Table 1**.

### Primary outcomes, secondary outcomes, and cycle characteristics of the overall sample in the ITT and PP populations

The clinical utility of PGT-A was evaluated in both the intention-to-treat (ITT) and per-protocol (PP) populations (**Tables 2**, **3**). Notably, in both the ITT and PP populations, baseline imbalances in key prognostic variables, including maternal age, paternal age, number of prior miscarriages, and antral follicle count (AFC), were observed between the PGT-A and IVF/ICSI groups, both in the overall sample and, to varying degrees, in subgroups stratified by age and number of miscarriages. To control for confounding bias, inverse probability of treatment weighting (IPTW) was applied separately to the two populations; the standardized mean differences (SMDs) for all covariates after weighting are presented in **Supplementary Table 2**. To ensure robustness, covariates with SMD > 0.1 were included in subsequent multivariable models. Descriptive results of the propensity score analyses are presented in **Supplementary Table 3**, and the propensity score density plots are shown in **Supplementary Figures 1** and **2**. Notably, one patient in the IVF/ICSI group had a pre-truncation stabilized weight of 62.41, corresponding to an estimated probability of PGT-A assignment close to 0.99—that is, a region of near-non-overlap between the treatment groups; truncation at the 1st and 99th percentiles bounded the influence of this observation (post-truncation weight 3.64; **Supplementary Table 3**).

**Table 2:** Baseline characteristics, cumulative live birth rate, and adjusted estimates in the PGT-A and conventional IVF/ICSI groups in the intention-to-treat population.

Characteristics	PGT-A group (n=342)	IVF/ICSI group (n=247)	*P*-value	P for interaction (age strata × group)	P for interaction (miscarriage strata × group)
Maternal age (years)	32.00 (29.25, 36.00)	34.00 (30.00, 38.00)	**0.004**		
Paternal age (years)	33.00 (30.00, 37.00)	35.00 (31.00, 39.00)	**0.001**		
BMI (kg/m2)	22.10 (20.40, 24.48)	22.60 (20.60, 25.00)	0.161		
Duration of infertility(years)	2.00 (1.00, 3.00)	2.00 (1.00, 4.00)	**< 0.001**		
Number of miscarriages	3.00 (2.00, 3.00)	2.00 (2.00, 2.00)	**< 0.001**		
AFC	13.00 (8.00, 18.00)	10.00 (5.00, 16.00)	**< 0.001**		
bFSH (IU/L)	6.74 (5.72, 8.13)	7.16 (5.79, 8.97)	0.135		
bE2 (pmol/L)	128.10 (91.88, 188.00)	150.00 (98.00, 219.00)	**0.013**		
bLH (mIU/mL)	4.68 (3.40, 6.08)	4.30 (3.09, 5.81)	**0.039**		
Infertility factor (%, n)					
Female factor	21.1% (72/342)	45.7% (113/247)	**< 0.001**		
Male factor	19.0% (65/342)	14.6% (36/247)	0.159		
Combined factors	13.2% (45/342)	6.9% (17/247)	**0.014**		
Others	46.8% (160/342)	32.8% (81/247)	**< 0.001**		
Total Gn dose (IU)	2100.00 (1718.75, 2641.25)	2250.00 (1575.00, 3000.00)	0.241		
Total Gn days (d)	10.00 (9.00, 11.00)	10.00 (8.00, 11.00)	0.906		
Number of oocytes retrieved	13.00 (8.00, 19.00)	8.00 (3.00, 14.00)	**< 0.001**		
MII oocyte count	9.00 (6.00, 15.00)	5.00 (2.00, 10.00)	**< 0.001**		
2PN zygote count	7.00 (4.00, 12.00)	4.00 (2.00, 8.00)	**< 0.001**		
2PN cleaved embryo count	7.00 (4.00, 12.00)	4.00 (2.00, 8.00)	**<0.001**		
Transferable blastocyst formation rate, median (IQR)	0.64 (0.38, 0.83)	0.62 (0.33, 0.90)	0.502		
High-quality blastocyst rate, median (IQR)	0.53 (0.25, 0.73)	0.50 (0.00, 0.78)	0.122		

Cumulative Transfer	PGT-A group (n=342)	IVF/ICSI group (n=247)	*P*-value		
Conservative Cumulative live birth	48.5% (166/342)	47.4% (117/247)	0.779		
Optimistic cumulative live birth rate	53.2% (182/342)	59.9% (148/247)	0.106		
Logistic Regression Model	aOR (95% CI)				
Conservative cumulative live birth rate	0.862 (0.591~1.255)		0.437	0.871	0.695
Optimistic cumulative live birth rate	0.691 (0.470~1.017)		0.061	0.454	0.916

IPTW: Maternal age (years), Paternal age (years), Duration of infertility(years), Number of miscarriages, AFC, bE2 (pmol/L), bLH (mIU/mL), Female factor, Combined factors, Others.

Adjusted: BMI (kg/m2).

Miscarriage strata × treatment interaction P values are exploratory: near-violation of the positivity assumption in the >2-miscarriage stratum renders the underlying stratum-specific coefficients—and therefore their contrast—unstable; these tests should not be interpreted as evidence of effect modification. Bold P values indicate statistical significance (P < 0.05).

**Table 3:** Baseline characteristics and pregnancy outcomes of the PGT-A and conventional IVF/ICSI groups in the per-protocol population.

Characteristics	PGT-A group (n=221)	IVF/ICSI group (n=168)	*P*-value	P for interaction (age strata × group)	P for interaction (miscarriage strata × group)
Maternal age (years)	32.00 (29.00, 34.00)	32.50 (30.00, 37.00)	**0.009**		
Paternal age (years)	32.00 (30.00, 35.50)	34.00 (31.00, 38.00)	**0.002**		
BMI (kg/m2)	22.00 (20.20, 24.20)	22.40 (20.60, 25.00)	0.055		
Duration of infertility(years)	1.00 (1.00, 2.00)	2.00 (1.00, 3.00)	**< 0.001**		
Number of miscarriages	3.00 (2.00, 3.00)	2.00 (2.00, 2.00)	**< 0.001**		
AFC	14.00 (11.00, 18.00)	11.50 (6.25, 18.00)	**< 0.001**		
bFSH (IU/L)	6.54 (5.61, 7.78)	6.57 (5.58, 8.85)	0.312		
bE2 (pmol/L)	124.85 (79.15, 182.00)	142.44 (83.92, 193.25)	0.229		
bLH (mIU/mL)	4.78 (3.58, 6.08)	4.43 (3.13, 6.01)	0.097		
Infertility factor (%, n)					
Female factor	22.2% (49/221)	47.0% (79/168)	**< 0.001**		
Male factor	20.8% (46/221)	13.7% (23/168)	0.068		
Combined factors	11.8% (26/221)	2.4% (4/168)	**< 0.001**		
Others	45.2% (100/221)	36.9% (62/168)	0.098		
Total Gn dose (IU)	2025.00 (1650.00, 2475.00)	2250.00 (1675.00, 3000.00)	**0.018**		
Total Gn days (d)	10.00 (9.00, 11.00)	10.00 (9.00, 12.00)	0.989		
First-cycle Endometrial thickness at the day of embryo transfer (mm)	9.60 (8.70, 11.00)	10.00 (9.10, 11.45)	0.108		
Number of oocytes retrieved	15.00 (10.00, 20.00)	10.00 (5.00, 15.00)	**< 0.001**		
MII oocyte count	12.00 (8.00, 16.00)	6.00 (3.00, 11.00)	**< 0.001**		
2PN zygote count	9.00 (6.00, 12.50)	5.00 (3.00, 9.00)	**< 0.001**		
2PN cleaved embryo count	8.00 (6.00, 12.50)	5.00 (3.00, 9.00)	**<0.001**		
Transferable blastocyst formation rate, median (IQR)	73.68% (57.14%, 91.99%)	70.32% (50.00%, 100.00%)	0.682		
High-quality blastocyst rate, median (IQR)	60.00% (43.30%, 80.00%)	60.00% (33.33%, 85.71%)	0.426		
First-cycle Stage of embryo transfer (%, n)					
Day5	77.8% (172/221)	83.3% (140/168)	0.177		
Day6	20.4% (45/221)	16.7% (28/168)	0.355		
Day7	1.8% (4/221)	0.0% (0/168)	0.137		
First-cycle ICM score of embryo transfer (%, n)			1.000		
A+B	99.1% (219/221)	99.4% (167/168)			
C	0.9% (2/221)	0.6% (1/168)			
First-cycle Trophectoderm score of embryo transfer (%, n)			0.574		
A+B	96.4% (213/221)	95.2% (160/168)			
C	3.6% (8/221)	4.8% (8/168)			

First Transfer	PGT-A group (n=221)	IVF/ICSI group (n=168)	*P*-value		
Biochemical pregnancy rate	71.9% (159/221)	67.9% (114/168)	0.383		
Clinical pregnancy rate	67.0% (148/221)	61.9% (104/168)	0.300		
Miscarriage rate^a^	4.7% (7/148)	5.8% (6/104)	0.713		
Live birth rate	63.8% (141/221)	58.3% (98/168)	0.272		
Logistic Regression Model	aOR (95% CI)				
Biochemical pregnancy rate	0.995 (0.580~1.706)		0.984	**0.006**	0.056
Clinical pregnancy rate	1.011 (0.607~1.684)		0.967	**0.004**	0.120
Live birth rate	0.942 (0.570~1.556)		0.814	**0.040**	0.135

Cumulative Transfer	PGT-A group (n=221)	IVF/ICSI group (n=168)	*P*-value		
Cumulative live birth	75.1% (166/221)	69.6% (117/168)	0.230		
Logistic Regression Model	aOR (95% CI)				
Cumulative live birth	0.858 (0.487~1.512)		0.595	0.071	0.289

Per Transfer	PGT-A group (n=268)	IVF/ICSI group (n=214)	*P*-value		
Biochemical pregnancy rate	72.0% (193/268)	65.0% (139/214)	0.097		
Clinical pregnancy rate	66.0% (177/268)	59.8% (128/214)	0.158		
Miscarriage rate^a^	6.3% (11/176)	8.6% (11/128)	0.436		
Live birth rate	61.9% (166/268)	54.7% (117/214)	0.107		
Generalized Estimating Equations	aOR (95% CI)				
Biochemical pregnancy rate	1.112 (0.685~1.806)		0.667	**0.011**	**0.010**
Clinical pregnancy rate	1.055 (0.670~1.661)		0.818	**0.010**	0.119
Live birth rate	0.980 (0.630~1.525)		0.929	0.064	0.094

IPTW: Maternal age (years), Paternal age (years), Duration of infertility(years), Number of miscarriages, AFC, Female factor, Combined factors, Total Gn dose (IU), Number of oocytes retrieved.

Adjusted: BMI (kg/m2), bE2 (pmol/L), Female factor.

^a^Miscarriage rate was calculated per intrauterine clinical pregnancy, with ectopic pregnancies excluded from the denominator.

Miscarriage strata × treatment interaction P values are exploratory: near-violation of the positivity assumption in the >2-miscarriage stratum renders the underlying stratum-specific coefficients—and therefore their contrast—unstable; these tests should not be interpreted as evidence of effect modification. Bold P values indicate statistical significance (P < 0.05).

In the ITT population, the PGT-A group (n=342) and the conventional IVF/ICSI group (n=247) differed significantly across several key baseline variables: patients in the PGT-A group were younger (median 32.0 vs. 34.0 years, P = 0.004), had a shorter duration of infertility (median 2.0 [IQR 1.0–3.0] vs. 2.0 [1.0–4.0] years, P<0.001), had more prior miscarriages (median 3.0 vs. 2.0, P<0.001), and had a higher antral follicle count (AFC) (13.0 vs. 10.0, P<0.001). The PGT-A group also had significantly higher numbers of oocytes retrieved (13.0 vs. 8.0), MII oocytes (9.0 vs. 5.0), and 2PN zygotes (7.0 vs. 4.0) compared with the control group (all P<0.001). No statistically significant differences were observed between the two groups in the transferable blastocyst formation rate (0.64 vs. 0.62, P = 0.502) or the high-quality blastocyst rate (0.53 vs. 0.50, P = 0.122).

In the ITT population, the conservative cumulative live birth rate was 48.5% (166/342) in the PGT-A group and 47.4% (117/247) in the IVF/ICSI group, with no statistically significant difference (P = 0.779). After adjustment using inverse probability of treatment weighting (IPTW), the adjusted odds ratio (aOR) for the conservative cumulative live birth rate was 0.862 (95% CI 0.591–1.255, P = 0.437). The optimistic cumulative live birth rate, assuming that all censored patients with remaining cryopreserved embryos eventually achieved a live birth, was 53.2% (182/342) in the PGT-A group and 59.9% (148/247) in the IVF/ICSI group, with an aOR of 0.691 (95% CI 0.470–1.017, P = 0.061). In the full covariate-adjusted model, the interaction P-values were 0.871 for age subgroup × treatment group and 0.695 (exploratory; see Statistical analysis) for miscarriage number subgroup × treatment group, neither of which reached statistical significance, indicating that neither age nor prior miscarriage number significantly modified the effect of PGT-A on ITT cumulative live birth rate.

In the per-protocol (PP) population, patients in the PGT-A group were younger (32.0 vs. 32.5 years, P = 0.009), had a shorter duration of infertility (1.0 vs. 2.0 years, P<0.001), had more prior miscarriages (3.0 vs. 2.0, P<0.001), and had significantly higher numbers of oocytes retrieved (15.0 vs. 10.0) and MII oocytes (12.0 vs. 6.0) (all P<0.001). The total gonadotropin dose was lower in the PGT-A group (2025 IU vs. 2250 IU, P = 0.018). No significant differences were observed between the two groups in blastocyst developmental stage (D5/D6/D7) or inner cell mass/trophectoderm scores at first transfer (all P>0.05). The live birth rate at first transfer was 63.8% (141/221) in the PGT-A group and 58.3% (98/168) in the control group, with no statistically significant difference (P = 0.272); the adjusted aOR was 0.942 (95% CI 0.570–1.556, P = 0.814). Secondary outcomes at first transfer also showed no significant differences: biochemical pregnancy rate (71.9% vs. 67.9%, P = 0.383; aOR 0.995, 95% CI 0.580–1.706, P = 0.984), clinical pregnancy rate (67.0% vs. 61.9%, P = 0.300; aOR 1.011, 95% CI 0.607–1.684, P = 0.967), and miscarriage rate (calculated per intrauterine clinical pregnancy: 4.7% vs. 5.8%, P = 0.713). In the PP population, the cumulative live birth rate was 75.1% (166/221) in the PGT-A group and 69.6% (117/168) in the IVF/ICSI group, with no statistically significant difference (P = 0.230); the adjusted aOR was 0.858 (95% CI 0.487–1.512, P = 0.595). The interaction P-values for PP cumulative live birth were 0.071 for age strata × treatment group and 0.289 (exploratory; see Statistical analysis) for miscarriage strata × treatment group, neither reaching statistical significance. Per-transfer cycle analysis included 268 cycles in the PGT-A group and 214 cycles in the IVF/ICSI group. Cycles contributed by the 16 PGT-A and 31 IVF/ICSI window-censored patients with remaining cryopreserved embryos were not included in the per-transfer analysis. No significant differences were observed between the two groups in biochemical pregnancy rate (72.0% vs. 65.0%, P = 0.097), clinical pregnancy rate (66.0% vs. 59.8%, P = 0.158), or live birth rate (61.9% vs. 54.7%, P = 0.107). In the sensitivity analysis using generalized estimating equations (GEE) to account for repeated measures across cycles, no statistically significant differences were found between the two groups for biochemical pregnancy (aOR 1.112, 95% CI 0.685–1.806, P = 0.667), clinical pregnancy (aOR 1.055, 95% CI 0.670–1.661, P = 0.818), or live birth (aOR 0.980, 95% CI 0.630–1.525, P = 0.929). In the GEE models, the age subgroup × treatment group interaction was significant for biochemical pregnancy (P = 0.011) and clinical pregnancy (P = 0.010) and borderline for live birth (P = 0.064); the miscarriage number subgroup × treatment group interaction was significant for biochemical pregnancy (P = 0.010) but not for clinical pregnancy (P = 0.119) or live birth (P = 0.094). These interaction signals—particularly those involving miscarriage strata, which are exploratory for the positivity reasons described in Statistical analysis—should be interpreted with caution and not as evidence of effect heterogeneity.

### Primary outcomes, secondary outcomes, and cycle characteristics stratified by age

#### For patients aged <35 years

In the ITT population, for the primary outcome, the conservative cumulative live birth rate was 55.3% (130/235) in the PGT-A group and 61.8% (84/136) in the IVF/ICSI group, with no statistically significant difference (P = 0.226) (**Tables 4**, **5**). After IPTW adjustment, the aOR for the PGT-A group was 0.791 (95% CI 0.481–1.303, P = 0.357). The optimistic cumulative live birth rate was 62.1% (146/235) in the PGT-A group and 72.8% (99/136) in the IVF/ICSI group (P = 0.037), with an adjusted aOR of 0.712 (95% CI 0.415–1.222, P = 0.218).

**Table 4:** Baseline characteristics, cumulative live birth rate, and adjusted estimates of the PGT-A and conventional IVF/ICSI groups in the intention-to-treat population, stratified by maternal age.

Characteristics	Age <35		*P*-value	*P*-value^b^	Age ≥35		*P*-value	*P*-value^b^
	PGT-A group (n=235)	IVF/ICSI group (n=136)			PGT-A group (n=107)	IVF/ICSI group (n=111)		
Maternal age (years)	31.00 (29.00, 32.00)	31.00 (28.75, 32.00)	0.772		39.00 (36.00, 41.00)	39.00 (37.00, 42.00)	0.378	
Paternal age (years)	32.00 (29.00, 33.00)	31.00 (29.00, 34.00)	0.870		39.00 (36.00, 42.00)	39.00 (37.00, 43.00)	0.208	
BMI (kg/m2)	21.60 (20.20, 24.00)	22.25 (20.48, 25.40)	0.051		23.20 (21.20, 25.20)	22.80 (20.70, 24.45)	0.340	
Duration of infertility(years)	1.00 (1.00, 2.00)	2.00 (1.00, 3.00)	**<0.001**		2.00 (1.00, 3.00)	2.00 (1.00, 4.00)	**0.005**	
Number of miscarriages	2.00 (2.00, 3.00)	2.00 (2.00, 2.00)	**<0.001**		3.00 (2.00, 3.00)	2.00 (2.00, 2.00)	**<0.001**	
AFC	14.00 (11.00, 19.00)	12.00 (7.00, 20.00)	**0.035**		9.00 (5.50, 16.00)	7.00 (3.00, 11.50)	**0.003**	
bFSH (IU/L)	6.65 (5.60, 7.78)	7.03 (5.78, 8.63)	0.196		7.32 (5.93, 9.30)	7.33 (5.87, 9.91)	0.777	
bE2 (pmol/L)	127.00 (94.06, 184.50)	147.50 (89.73, 195.07)	0.198		136.00 (89.64, 202.00)	161.00 (108.50, 247.00)	0.078	
bLH (mIU/mL)	4.84 (3.74, 6.12)	4.48 (3.45, 6.04)	0.170		4.15 (3.20, 5.91)	4.24 (2.74, 5.20)	0.344	
Infertility factor (%, n)								
Female factor	21.7% (51/235)	47.1% (64/136)	**< 0.001**		19.6% (21/107)	44.1% (49/111)	**< 0.001**	
Male factor	18.3% (43/235)	18.4% (25/136)	0.984		20.6% (22/107)	9.9% (11/111)	**0.028**	
Combined factors	10.6% (25/235)	4.4% (6/136)	**0.037**		18.7% (20/107)	9.9% (11/111)	0.063	
Others	49.4% (116/235)	30.1% (41/136)	**< 0.001**		41.1% (44/107)	36.0% (40/111)	0.441	
Total Gn dose (IU)	2025.00 (1650.00, 2475.00)	1950.00 (1500.00, 2643.75)	0.482		2250.00 (1800.00, 2925.00)	2700.00 (1800.00, 3300.00)	0.089	
Total Gn days (d)	10.00 (9.00, 11.00)	10.00 (8.00, 12.00)	0.455		10.00 (9.00, 11.00)	10.00 (8.00, 12.00)	0.514	
Number of oocytes retrieved	14.00 (10.00, 20.00)	11.00 (6.00, 17.00)	**< 0.001**		9.00 (4.00, 14.50)	5.00 (3.00, 10.00)	**0.002**	
MII oocyte count	11.00 (7.00, 15.00)	7.00 (3.00, 11.25)	**< 0.001**		7.00 (3.00, 11.50)	4.00 (1.00, 7.00)	**< 0.001**	
2PN zygote count	8.00 (5.00, 12.00)	5.00 (3.00, 10.25)	**< 0.001**		5.00 (2.00, 9.00)	3.00 (1.00, 5.00)	**0.001**	
2PN cleaved embryo count	8.00 (5.00, 12.00)	5.00 (3.00, 10.00)	**< 0.001**		5.00 (2.00, 9.00)	3.00 (1.00, 6.00)	**0.003**	
Transferable blastocyst formation rate, median (IQR)	0.67 (0.46, 0.87)	0.62 (0.40, 0.86)	0.178		0.50 (0.00, 0.75)	0.60 (0.17, 1.00)	0.181	
High-quality blastocyst rate, median (IQR)	0.57 (0.33, 0.75)	0.50 (0.32, 0.80)	0.295		0.43 (0.00, 0.63)	0.33 (0.00, 0.72)	0.754	

Cumulative transfer	PGT-A group (n=235)	IVF/ICSI group (n=136)	*P*-value	*P*-value^b^	PGT-A group (n=107)	IVF/ICSI group (n=111)	*P*-value	*P*-value^b^
Conservative cumulative live birth rate	55.3% (130/235)	61.8% (84/136)	0.226		33.6% (36/107)	29.7% (33/111)	0.534	
Optimistic cumulative live birth rate	62.1% (146/235)	72.8% (99/136)	**0.037**		33.6% (36/107)	44.1% (49/111)	0.112	
Logistic Regression Model	aOR (95% CI)				aOR (95% CI)			
Conservative cumulative live birth rate	0.791 (0.481~1.303)		0.357	0.714	0.846 (0.447~1.603)		0.608	1.000
Optimistic cumulative live birth rate	0.712 (0.415~1.222)		0.218	0.435	0.520 (0.280~0.966)		0.039	0.077

IPTW: Maternal age (years), Paternal age (years), Duration of infertility(years), Number of miscarriages, AFC, bE2 (pmol/L), bLH (mIU/mL), Female factor, Combined factors, Others.

Adjusted: BMI (kg/m2).

^b^P values were Bonferroni-corrected for two comparisons (m = 2; one comparison in each of the two strata of the subgrouping variable), with a corrected significance level of α’ = 0.025. Corrected P values were calculated as nominal P × 2, and values exceeding 1 were reported as 1.000.

Bold P values indicate statistical significance (P < 0.05).

**Table 5:** Baseline characteristics and pregnancy outcomes of the PGT-A and conventional IVF/ICSI groups in the per-protocol population, stratified by maternal age.

Characteristics	Age <35		*P*-value	*P*-value^b^	Age ≥35		*P*-value	*P*-value^b^
	PGT-A group (n=169)	IVF/ICSI group (n=103)			PGT-A group (n=52)	IVF/ICSI group (n=65)		
Maternal age (years)	31.00 (29.00, 32.00)	31.00 (29.00, 32.00)	0.820		37.50 (35.00, 39.00)	37.00 (36.00, 40.00)	0.328	
Paternal age (years)	31.00 (29.00, 33.00)	31.00 (30.00, 33.00)	0.546		38.00 (35.75, 40.00)	39.00 (37.00, 41.00)	0.217	
BMI (kg/m2)	21.60 (20.10, 23.40)	22.30 (20.65, 25.20)	**0.014**		23.10 (21.08, 25.38)	22.50 (20.60, 24.60)	0.400	
Duration of infertility(years)	1.00 (1.00, 2.00)	2.00 (1.00, 3.00)	**0.012**		1.00 (1.00, 2.00)	2.00 (1.00, 4.00)	**0.008**	
Number of miscarriages	2.00 (2.00, 2.00)	2.00 (2.00, 3.00)	**< 0.001**		3.00 (2.00, 3.00)	2.00 (2.00, 2.00)	**< 0.001**	
AFC	14.00 (12.00, 19.00)	13.00 (8.00, 21.00)	0.085		13.00 (8.00, 17.00)	8.00 (5.00, 13.00)	**<0.001**	
bFSH (IU/L)	6.59 (5.58, 7.76)	6.43 (5.51, 8.19)	0.755		6.39 (5.72, 7.96)	6.69 (5.80, 9.23)	0.291	
bE2 (pmol/L)	125.27 (84.00, 179.00)	138.00 (81.65, 185.00)	0.685		118.50 (60.88, 206.00)	144.00 (93.63, 233.00)	0.258	
bLH (mIU/mL)	4.91 (3.88, 6.04)	4.49 (3.51, 6.13)	0.265		4.53 (3.20, 6.49)	4.30 (2.70, 5.63)	0.422	
Infertility factor (%, n)								
Female factor	22.5% (38/169)	48.5% (50/103)	**< 0.001**		21.2% (11/52)	44.6% (29/65)	**0.008**	
Male factor	20.7% (35/169)	17.5% (18/103)	0.514		21.2% (11/52)	7.7% (5/65)	**0.035**	
Combined factors	11.8% (20/169)	1.9% (2/103)	**0.004**		11.5% (6/52)	3.1% (2/65)	0.152	
Others	45.0% (76/169)	32.0% (33/103)	**0.035**		46.2% (24/52)	44.6% (29/65)	0.868	
Total Gn dose (IU)	2025.00 (1650.00, 2400.00)	1950.00 (1500.00, 2700.00)	0.786		2225.00 (1800.00, 3000.00)	2700.00 (2100.00, 3375.00)	**0.037**	
Total Gn days (d)	10.00 (9.00, 11.00)	10.00 (9.00, 12.00)	0.904		10.00 (9.00, 11.00)	10.00 (9.00, 12.00)	0.862	
First-cycle Endometrial thickness at the day of embryo transfer (mm)	9.50 (8.70, 11.00)	10.00 (9.20, 11.75)	**0.002**		9.90 (8.80, 11.00)	10.20 (9.00, 11.00)	0.420	
Number of oocytes retrieved	15.00 (11.00, 20.00)	12.00 (7.00, 18.00)	**< 0.001**		13.00 (8.00, 18.25)	8.00 (3.00, 12.00)	**< 0.001**	
MII oocyte count	12.00 (8.00, 16.00)	7.00 (4.00, 13.00)	**< 0.001**		10.50 (6.00, 15.00)	5.00 (2.00, 8.00)	**< 0.001**	
2PN zygote count	9.00 (6.00, 13.00)	6.00 (3.00, 12.00)	**< 0.001**		8.00 (5.75, 12.00)	3.00 (2.00, 6.00)	**< 0.001**	
2PN cleaved embryo count	9.00 (6.00, 13.00)	6.00 (3.00, 11.50)	**< 0.001**		8.00 (5.75, 12.00)	3.00 (2.00, 6.00)	**< 0.001**	
Transferable blastocyst formation rate, median (IQR)	0.73 (0.56, 0.93)	0.67 (0.50, 0.90)	0.343		0.75 (0.60, 0.91)	0.80 (0.50, 1.00)	0.547	
High-quality blastocyst rate, median (IQR)	0.60 (0.45, 0.80)	0.60 (0.40, 0.85)	0.717		0.60 (0.43, 0.78)	0.60 (0.29, 1.00)	0.461	
First-cycle Stage of embryo transfer (%, n)								
Day5	80.5% (136/169)	86.4% (89/103)	0.209		69.2% (36/52)	78.5% (51/65)	0.256	
Day6	17.8% (30/169)	13.6% (14/103)	0.366		28.8% (15/52)	21.5% (14/65)	0.363	
Day7	1.8% (3/169)	0.0% (0/103)	0.292		1.9% (1/52)	0.0% (0/65)	0.444	
First-cycle ICM score of embryo transfer (%, n)			1.000				1.000	
A+B	99.4% (168/169)	100.0% (103/103)			98.1% (51/52)	98.5% (64/65)		
C	0.6% (1/169)	0.0% (0/103)			1.9% (1/52)	1.5% (1/65)		
First-cycle Trophectoderm score of embryo transfer (%, n)			0.511				0.530	
A+B	97.0% (164/169)	99.0% (102/103)			94.2% (49/52)	89.2% (58/65)		
C	3.0% (5/169)	1.0% (1/103)			5.8% (3/52)	10.8% (7/65)		

First transfer	PGT-A group (n=169)	IVF/ICSI group (n=103)	*P*-value	*P*-value^b^	PGT-A group (n=52)	IVF/ICSI group (n=65)	*P*-value	*P*-value^b^
Biochemical pregnancy rate	72.2% (122/169)	79.6% (82/103)	0.170		71.2% (37/52)	49.2% (32/65)	**0.017**	
Clinical pregnancy rate	66.3% (112/169)	73.8% (76/103)	0.193		69.2% (36/52)	43.1% (28/65)	**0.005**	
Miscarriage rate^a^	3.6% (4/112)	7.9% (6/76)	0.195		8.3% (3/36)	0.0% (0/28)	0.250	
Live birth rate	63.9% (108/169)	68.0% (70/103)	0.495		63.5% (33/52)	43.1% (28/65)	**0.028**	
Logistic Regression Model	aOR (95% CI)		*P*-Value	*P*-Value ^b^	aOR (95% CI)		*P*-Value	*P*-Value ^b^
Biochemical pregnancy rate	0.516 (0.256~1.042)		0.065	0.130	2.657 (1.049~6.730)		0.039	0.079
Clinical pregnancy rate	0.565 (0.297~1.073)		0.081	0.162	2.873 (1.156~7.144)		0.023	0.047
Live birth rate	0.639 (0.344~1.186)		0.155	0.311	1.932 (0.818~4.561)		0.133	0.265

Cumulative transfer	PGT-A group (n=169)	IVF/ICSI group (n=103)	*P*-value	*P*-value ^b^	PGT-A group (n=52)	IVF/ICSI group (n=65)	*P*-value	*P*-value ^b^
Cumulative live birth	76.9% (130/169)	81.6% (84/103)	0.366		69.2% (36/52)	50.8% (33/65)	**0.044**	
Logistic Regression Model	aOR (95% CI)				aOR (95% CI)			
Cumulative live birth	0.531 (0.249~1.132)		0.101	0.202	1.543 (0.639~3.728)		0.334	0.669

Per transfer	PGT-A group (n=207)	IVF/ICSI group (n=130)	*P*-value		PGT-A group (n=61)	IVF/ICSI group (n=84)	*P*-value	
Biochemical pregnancy rate	73.4% (152/207)	77.7% (101/130)	0.379		67.2% (41/61)	45.2% (38/84)	**0.009**	
Clinical pregnancy rate	66.7% (138/207)	72.3% (94/130)	0.276		63.9% (39/61)	40.5% (34/84)	**0.005**	
Miscarriage rate^a^	5.8% (8/138)	10.6% (10/94)	0.176		7.9% (3/38)	2.9% (1/34)	0.360	
Live birth rate	62.8% (130/207)	64.6% (84/130)	0.736		59.0% (36/61)	39.3% (33/84)	**0.019**	

Generalized estimating equations	aOR (95% CI)		*P*-value	*P*-value ^b^	aOR (95% CI)		*P*-value	*P*-value ^b^
Biochemical pregnancy rate	0.629 (0.334~1.184)		0.151	0.301	2.483 (1.067~5.781)		**0.035**	0.070
Clinical pregnancy rate	0.642 (0.360~1.143)		0.132	0.263	2.439 (1.071~5.557)		**0.034**	0.068
Live birth rate	0.709 (0.409~1.229)		0.220	0.440	1.725 (0.802~3.709)		0.162	0.324

IPTW: Maternal age (years), Paternal age (years), Duration of infertility(years), Number of miscarriages, AFC, Female factor, Combined factors, Total Gn dose (IU), Number of oocytes retrieved.

Adjusted: BMI (kg/m2), bE2 (pmol/L), Female factor.

^a^ Miscarriage rate was calculated per intrauterine clinical pregnancy, with ectopic pregnancies excluded from the denominator.

^b^ P values were Bonferroni-corrected for two comparisons (m = 2; one comparison in each of the two strata of the subgrouping variable), with a corrected significance level of α’ = 0.025. Corrected P values were calculated as nominal P × 2, and values exceeding 1 were reported as 1.000. Bold P values indicate statistical significance (P < 0.05).

In the PP population, for first-transfer outcomes, the biochemical pregnancy rate was 72.2% (122/169) in the PGT-A group and 79.6% (82/103) in the IVF/ICSI group (P = 0.170), with an adjusted aOR of 0.516 (95% CI 0.256–1.042, P = 0.065; Bonferroni-corrected P = 0.130). The clinical pregnancy rate was 66.3% (112/169) in the PGT-A group and 73.8% (76/103) in the IVF/ICSI group (P = 0.193), with an adjusted aOR of 0.565 (95% CI 0.297–1.073, P = 0.081; Bonferroni-corrected P = 0.162). The miscarriage rate was 3.6% (4/112) in the PGT-A group and 7.9% (6/76) in the IVF/ICSI group (P = 0.195). The live birth rate was 63.9% (108/169) in the PGT-A group and 68.0% (70/103) in the IVF/ICSI group (P = 0.495), with an adjusted aOR of 0.639 (95% CI 0.344–1.186, P = 0.155; Bonferroni-corrected P = 0.311). The cumulative live birth rate was 76.9% (130/169) in the PGT-A group and 81.6% (84/103) in the IVF/ICSI group (P = 0.366), with an adjusted aOR of 0.531 (95% CI 0.249–1.132, P = 0.101; Bonferroni-corrected P = 0.202). In the GEE sensitivity analysis, among patients aged <35 years, no significant differences were observed between PGT-A and IVF/ICSI for any outcome: biochemical pregnancy aOR 0.629 (95% CI 0.334–1.184, P = 0.151; Bonferroni-corrected P = 0.301), clinical pregnancy aOR 0.642 (95% CI 0.360–1.143, P = 0.132; Bonferroni-corrected P = 0.263), and live birth aOR 0.709 (95% CI 0.409–1.229, P = 0.220; Bonferroni-corrected P = 0.440).

#### For patients aged ≥35 years

In the ITT population, for the primary outcome, the conservative cumulative live birth rate was 33.6% (36/107) in the PGT-A group and 29.7% (33/111) in the IVF/ICSI group, with no statistically significant difference (P = 0.534). After IPTW adjustment, the aOR was 0.846 (95% CI 0.447–1.603, P = 0.608; Bonferroni-corrected P = 1.000). The optimistic cumulative live birth rate was 33.6% (36/107) in the PGT-A group and 44.1% (49/111) in the IVF/ICSI group (P = 0.112), with an adjusted aOR of 0.520 (95% CI 0.280–0.966, P = 0.039; Bonferroni-corrected P = 0.077).

In the PP population, for first-transfer outcomes, the biochemical pregnancy rate was 71.2% (37/52) in the PGT-A group and 49.2% (32/65) in the IVF/ICSI group (P = 0.017), with an adjusted aOR of 2.657 (95% CI 1.049–6.730, P = 0.039; Bonferroni-corrected P = 0.079). The clinical pregnancy rate was 69.2% (36/52) in the PGT-A group and 43.1% (28/65) in the IVF/ICSI group (P = 0.005), with an adjusted aOR of 2.873 (95% CI 1.156–7.144, P = 0.023; Bonferroni-corrected P = 0.047). Given the small subgroup sample size and the potential for chance findings from multiple testing, this result should be interpreted cautiously. The miscarriage rate was 8.3% (3/36) in the PGT-A group and 0.0% (0/28) in the IVF/ICSI group (P = 0.250). The live birth rate was 63.5% (33/52) in the PGT-A group and 43.1% (28/65) in the IVF/ICSI group (P = 0.028), with an adjusted aOR of 1.932 (95% CI 0.818–4.561, P = 0.133; Bonferroni-corrected P = 0.265). The cumulative live birth rate was 69.2% (36/52) in the PGT-A group and 50.8% (33/65) in the IVF/ICSI group (P = 0.044), with an adjusted aOR of 1.543 (95% CI 0.639–3.728, P = 0.334). In the GEE sensitivity analysis, among patients aged ≥35 years, PGT-A was associated with a higher clinical pregnancy rate (aOR 2.439, 95% CI 1.071–5.557, P = 0.034; Bonferroni-corrected P = 0.068), biochemical pregnancy was nominally significant (aOR 2.483, 95% CI 1.067–5.781, P = 0.035) but not after Bonferroni correction (corrected P = 0.070), and live birth did not differ significantly (aOR 1.725, 95% CI 0.802–3.709, P = 0.162; Bonferroni-corrected P = 0.324). Across the two age strata, the age × treatment group interaction for first-transfer outcomes was significant for biochemical pregnancy (P = 0.006) and clinical pregnancy (P = 0.004) and nominally significant for live birth (P = 0.040) (**Table 3**).

### Primary outcomes, secondary outcomes, and cycle characteristics stratified by miscarriages

#### For patients with =2 miscarriages

In the ITT population, the conservative cumulative live birth rate in this subgroup was 53.8% (91/169) in the PGT-A group and 47.0% (93/198) in the control group, with no statistically significant difference (P = 0.189) (**Tables 6**, **7**). After IPTW adjustment, the aOR for conservative cumulative live birth in the PGT-A group was 0.930 (95% CI 0.595–1.455, P = 0.752; Bonferroni-corrected P = 1.000). The optimistic cumulative live birth rate was 58.6% (99/169) in the PGT-A group and 60.6% (120/198) in the control group, also without statistical significance (P = 0.693), with an adjusted aOR of 0.696 (95% CI 0.440–1.099, P = 0.120; Bonferroni-corrected P = 0.240).

**Table 6:** Baseline characteristics, cumulative live birth rate, and adjusted estimates of the PGT-A and conventional IVF/ICSI groups in the intention-to-treat population, stratified by the number of previous miscarriages.

Characteristics	Miscarriages =2		*P*-value	*P*-value^b^	Miscarriages >2		*P*-value	*P*-value^b^
	PGT-A group (n=169)	IVF/ICSI group (n=198)			PGT-A group (n=173)	IVF/ICSI group (n=49)		
Maternal age (years)	32.00 (29.00, 35.00)	34.00 (30.00, 38.00)	**0.003**		33.00 (30.00, 36.00)	34.00 (31.00, 39.00)	0.228	
Paternal age (years)	32.00 (30.00, 36.00)	34.00 (31.00, 39.00)	**< 0.001**		34.00 (31.00, 37.00)	37.00 (31.00, 39.00)	0.106	
BMI (kg/m2)	22.30 (20.80, 24.80)	22.30 (20.33, 24.80)	0.827		21.70 (20.20, 24.00)	23.10 (21.30, 25.80)	**0.004**	
Duration of infertility(years)	1.00 (1.00, 2.00)	2.00 (1.00, 4.00)	**< 0.001**		2.00 (1.00, 3.00)	2.00 (1.00, 3.00)	0.115	
Number of miscarriages	2.00 (2.00, 2.00)	2.00 (2.00, 2.00)	1.000		3.00 (3.00, 4.00)	3.00 (3.00, 3.00)	0.065	
AFC	14.00 (8.00, 18.00)	10.00 (5.00, 15.00)	**< 0.001**		13.00 (8.00, 17.00)	9.00 (4.00, 18.00)	**0.033**	
bFSH (IU/L)	6.94 (5.73, 8.68)	7.27 (5.84, 9.01)	0.531		6.59 (5.71, 7.87)	6.71 (5.45, 8.86)	0.567	
bE2 (pmol/L)	128.00 (89.13, 185.00)	147.50 (90.61, 218.75)	0.060		129.52 (95.00, 197.00)	166.00 (116.00, 199.00)	**0.028**	
bLH (mIU/mL)	4.64 (3.25, 6.18)	4.34 (3.20, 5.60)	0.133		4.71 (3.55, 5.97)	4.30 (3.06, 5.86)	0.152	
Infertility factor (%, n)								
Female factor	24.9% (42/169)	42.9% (85/198)	**< 0.001**		17.3% (30/173)	57.1% (28/49)	**< 0.001**	
Male factor	17.8% (30/169)	15.7% (31/198)	0.591		20.2% (35/173)	10.2% (5/49)	0.107	
Combined factors	13.0% (22/169)	7.1% (14/198)	0.056		13.3% (23/173)	6.1% (3/49)	0.168	
Others	44.4% (75/169)	34.3% (68/198)	**0.049**		49.1% (85/173)	26.5% (13/49)	**0.005**	
Total Gn dose (IU)	2125.00 (1750.00, 2575.00)	2250.00 (1565.62, 3000.00)	0.462		2100.00 (1700.00, 2700.00)	2250.00 (1575.00, 2812.50)	0.436	
Total Gn days (d)	10.00 (9.00, 12.00)	10.00 (8.00, 12.00)	0.632		10.00 (9.00, 11.00)	10.00 (9.00, 11.00)	0.897	
Number of oocytes retrieved	13.00 (7.00, 20.00)	8.00 (3.00, 14.00)	**< 0.001**		13.00 (9.00, 18.00)	7.00 (3.00, 12.00)	**< 0.001**	
MII oocyte count	9.00 (6.00, 15.00)	5.00 (2.00, 10.00)	**< 0.001**		9.00 (6.00, 14.00)	4.00 (2.00, 8.00)	**< 0.001**	
2PN zygote count	7.00 (4.00, 12.00)	4.00 (2.00, 8.00)	**< 0.001**		7.00 (4.00, 12.00)	3.00 (1.00, 6.00)	**< 0.001**	
2PN cleaved embryo count	7.00 (4.00, 12.00)	4.00 (2.00, 8.00)	**< 0.001**		7.00 (4.00, 11.00)	3.00 (1.00, 6.00)	**< 0.001**	
Transferable blastocyst formation rate, median (IQR)	0.62 (0.35, 0.86)	0.61 (0.33, 0.89)	0.587		0.64 (0.42, 0.80)	0.67 (0.29, 1.00)	0.929	
High-quality blastocyst rate, median (IQR)	0.53 (0.25, 0.75)	0.48 (0.00, 0.77)	0.171		0.53 (0.27, 0.71)	0.50 (0.00, 0.77)	0.575	

Cumulative transfer	PGT-A group (n=169)	IVF/ICSI group (n=198)	*P*-value	*P*-value^b^	PGT-A group (n=173)	IVF/ICSI group (n=49)	*P*-value	*P*-value^b^
Conservative cumulative live birth rate	53.8% (91/169)	47.0% (93/198)	0.189		43.4% (75/173)	49.0% (24/49)	0.484	
Optimistic cumulative live birth rate	58.6% (99/169)	60.6% (120/198)	0.693		48.0% (83/173)	57.1% (28/49)	0.257	
Logistic Regression Model	aOR (95% CI)				aOR (95% CI)			
Conservative cumulative live birth rate	0.930 (0.595~1.455)		0.752	1.000				
Optimistic cumulative live birth rate	0.696 (0.440~1.099)		0.120	0.240				

IPTW: Maternal age (years), Paternal age (years), Duration of infertility(years), Number of miscarriages, AFC, bE2 (pmol/L), bLH (mIU/mL), Female factor, Combined factors, Others.

Adjusted: BMI (kg/m2).

^b^ P values were Bonferroni-corrected for two comparisons (m = 2; one comparison in each of the two strata of the subgrouping variable), with a corrected significance level of α’ = 0.025. Corrected P values were calculated as nominal P × 2, and values exceeding 1 were reported as 1.000. Bold P values indicate statistical significance (P < 0.05).

**Table 7:** Baseline characteristics and pregnancy outcomes of the PGT-A and conventional IVF/ICSI groups in the per-protocol population, stratified by the number of previous miscarriages.

Characteristics	Miscarriages =2		*P*-value	*P*-value^b^	Miscarriages >2		*P*-value
	PGT-A group (n=110)	IVF/ICSI group (n=133)			PGT-A group (n=111)	IVF/ICSI group (n=35)	
Maternal age (years)	31.00 (29.00, 34.00)	32.00 (30.00, 37.00)	**0.014**		32.00 (30.00, 35.00)	33.00 (30.50, 36.00)	0.164
Paternal age (years)	32.00 (29.00, 35.00)	33.00 (31.00, 38.00)	**0.004**		33.00 (31.00, 36.00)	37.00 (31.00, 39.00)	**0.024**
BMI (kg/m2)	22.10 (20.42, 24.35)	22.10 (20.40, 24.80)	0.685		21.60 (20.10, 23.50)	23.40 (21.45, 26.00)	**0.002**
Duration of infertility(years)	1.00 (1.00, 2.00)	2.00 (1.00, 3.00)	**< 0.001**		1.00 (1.00, 3.00)	2.00 (1.00, 3.00)	0.076
Number of miscarriages	2.00 (2.00, 2.00)	2.00 (2.00, 2.00)	**1.000**		3.00 (3.00, 4.00)	3.00 (3.00, 3.00)	0.068
AFC	15.00 (12.00, 20.00)	11.00 (6.00, 17.00)	**< 0.001**		14.00 (11.00, 17.00)	12.00 (7.50, 20.00)	0.156
bFSH (IU/L)	6.71 (5.70, 7.98)	6.77 (5.79, 8.96)	0.562		6.32 (5.50, 7.68)	6.41 (5.21, 7.40)	0.792
bE2 (pmol/L)	126.00 (79.47, 184.00)	138.00 (77.07, 186.00)	0.564		123.51 (81.15, 179.50)	152.00 (109.00, 192.88)	0.113
bLH (mIU/mL)	4.68 (3.38, 6.07)	4.39 (3.31, 6.10)	0.236		4.83 (3.62, 6.02)	4.47 (3.16, 5.75)	0.223
Infertility factor (%, n)							
Female factor	27.3% (30/110)	42.9% (57/133)	**0.012**		17.1% (19/111)	62.9% (22/35)	**< 0.001**
Male factor	19.1% (21/110)	14.3% (19/133)	0.315		22.5% (25/111)	11.4% (4/35)	0.151
Combined factors	13.6% (15/110)	2.3% (3/133)	**< 0.001**		9.9% (11/111)	2.9% (1/35)	0.331
Others	40.0% (44/110)	40.6% (54/133)	0.924		50.5% (56/111)	22.9% (8/35)	**0.004**
Total Gn dose (IU)	2062.50 (1756.25, 2475.00)	2250.00 (1650.00, 3000.00)	0.113		2000.00 (1650.00, 2400.00)	2250.00 (1800.00, 2962.50)	0.161
Total Gn days (d)	10.00 (9.00, 11.00)	10.00 (9.00, 12.00)	0.550		10.00 (9.00, 11.00)	10.00 (9.00, 11.50)	0.694
First-cycle Endometrial thickness at the day of embryo transfer (mm)	9.60 (8.80, 11.00)	10.10 (9.10, 11.50)	**0.048**		9.60 (8.70, 10.55)	9.90 (9.10, 11.05)	0.111
Number of oocytes retrieved	15.00 (10.00, 21.00)	10.00 (5.00, 15.00)	**< 0.001**		15.00 (10.00, 19.00)	9.00 (5.50, 14.50)	**< 0.001**
MII oocyte count	11.50 (8.00, 16.00)	6.00 (3.00, 11.00)	**< 0.001**		12.00 (7.00, 15.00)	6.00 (3.00, 8.50)	**< 0.001**
2PN zygote count	8.50 (5.25, 13.00)	5.00 (3.00, 9.00)	**< 0.001**		9.00 (6.00, 12.00)	4.00 (2.00, 7.00)	**< 0.001**
2PN cleaved embryo count	8.00 (5.25, 13.00)	5.00 (3.00, 9.00)	**< 0.001**		9.00 (6.00, 12.00)	5.00 (2.00, 7.50)	**< 0.001**
Transferable blastocyst formation rate, median (IQR)	0.75 (0.56, 1.00)	0.69 (0.50, 1.00)	0.604		0.71 (0.59, 0.90)	0.75 (0.50, 1.00)	0.851
High-quality blastocyst rate, median (IQR)	0.61 (0.43, 0.80)	0.60 (0.33, 0.86)	0.471		0.60 (0.47, 0.80)	0.67 (0.37, 0.85)	0.762
First-cycle Stage of embryo transfer (%, n)							
Day5	81.8% (90/110)	82.7% (110/133)	0.857		73.9% (82/111)	85.7% (30/35)	0.148
Day6	16.4% (18/110)	17.3% (23/133)	0.847		24.3% (27/111)	14.3% (5/35)	0.211
Day7	1.8% (2/110)	0.0% (0/133)	0.204		1.8% (2/111)	0.0% (0/35)	1.000
**First-cycle ICM score of embryo transfer (%, n)**			1.000				1.000
A+B	100.0% (110/110)	99.2% (132/133)			98.2% (109/111)	100.0% (35/35)	
C	0.0% (0/110)	0.8% (1/133)			1.8% (2/111)	0.0% (0/35)	
First-cycle Trophectoderm score of embryo transfer (%, n)			0.986				0.952
A+B	96.4% (106/110)	95.5% (127/133)			96.4% (107/111)	94.3% (33/35)	
C	3.6% (4/110)	4.5% (6/133)			3.6% (4/111)	5.7% (2/35)	

First transfer	PGT-A group (n=110)	IVF/ICSI group (n=133)	*P*-value	*P*-value^b^	PGT-A group (n=111)	IVF/ICSI group (n=35)	*P*-value
Biochemical pregnancy rate	78.2% (86/110)	66.2% (88/133)	**0.039**		65.8% (73/111)	74.3% (26/35)	0.347
Clinical pregnancy rate	74.5% (82/110)	61.7% (82/133)	**0.033**		59.5% (66/111)	62.9% (22/35)	0.720
Miscarriage rate^a^	4.9% (4/82)	6.1% (5/82)	1.000		4.5% (3/66)	4.5% (1/22)	1.000
Live birth rate	70.9% (78/110)	57.9% (77/133)	**0.036**		56.8% (63/111)	60.0% (21/35)	0.735
Logistic Regression Model	aOR (95% CI)				aOR (95% CI)		
Biochemical pregnancy rate	1.570 (0.810~3.044)		0.181	0.362			
Clinical pregnancy rate	1.442 (0.766~2.713)		0.256	0.512			
Live birth rate	1.302 (0.706~2.402)		0.398	0.795			

Cumulative transfer	PGT-A group (n=110)	IVF/ICSI group (n=133)	*P*-value	*P*-value^b^	PGT-A group (n=111)	IVF/ICSI group (n=35)	*P*-value
Cumulative live birth	82.7% (91/110)	69.9% (93/133)	**0.021**		67.6% (75/111)	68.6% (24/35)	0.912
Logistic Regression Model	aOR (95% CI)				aOR (95% CI)		
Cumulative live birth	1.139 (0.564~2.300)		0.716	1.000			

Per transfer	PGT-A group (n=129)	IVF/ICSI group (n=172)	*P*-value	*P*-value^b^	PGT-A group (n=139)	IVF/ICSI group (n=42)	*P*-value
Biochemical pregnancy rate	79.8% (103/129)	62.8% (108/172)	**0.001**		64.7% (90/139)	73.8% (31/42)	0.274
Clinical pregnancy rate	75.2% (97/129)	59.9% (103/172)	**0.005**		57.6% (80/139)	59.5% (25/42)	0.821
Miscarriage rate^a^	6.2% (6/97)	9.7% (10/103)	0.359		6.3% (5/79)	4.0% (1/25)	0.663
Live birth rate	70.5% (91/129)	54.1% (93/172)	**0.004**		54.0% (75/139)	57.1% (24/42)	0.716
Generalized estimating equations	aOR (95% CI)				aOR (95% CI)		
Biochemical pregnancy rate	1.876 (1.028~3.424)		**0.041**	0.081			
Clinical pregnancy rate	1.501 (0.853~2.642)		0.158	0.317			
Live birth rate	1.365 (0.796~2.340)		0.258	0.515			

IPTW: Maternal age (years), Paternal age (years), Duration of infertility(years), Number of miscarriages, AFC, Female factor, Combined factors, Total Gn dose (IU), Number of oocytes retrieved.

Adjusted: BMI (kg/m2), bE2 (pmol/L), Female factor.

^a^ Miscarriage rate was calculated per intrauterine clinical pregnancy, with ectopic pregnancies excluded from the denominator.

^b^ P values were Bonferroni-corrected for two comparisons (m = 2; one comparison in each of the two strata of the subgrouping variable), with a corrected significance level of α’ = 0.025. Corrected P values were calculated as nominal P × 2, and values exceeding 1 were reported as 1.000. Bold P values indicate statistical significance (P < 0.05).

In the PP population, for first-transfer outcomes, the PGT-A group showed significantly higher biochemical pregnancy rates (78.2% vs. 66.2%, P = 0.039), clinical pregnancy rates (74.5% vs. 61.7%, P = 0.033), and live birth rates (70.9% vs. 57.9%, P = 0.036) compared with the control group. However, after IPTW and multivariable adjustment, the differences in biochemical pregnancy (aOR 1.570, 95% CI 0.810–3.044, P = 0.181), clinical pregnancy (aOR 1.442, 95% CI 0.766–2.713, P = 0.256), and live birth (aOR 1.302, 95% CI 0.706–2.402, P = 0.398) were no longer statistically significant (Bonferroni-corrected P-values were 0.362, 0.512 and 0.795 respectively). The conservative cumulative live birth rate was 82.7% (91/110) in the PGT-A group and 69.9% (93/133) in the control group, with a statistically significant difference (P = 0.021); however, after multivariable adjustment, the aOR was 1.139 (95% CI 0.564–2.300, P = 0.716), and the corrected P-value was 1.000, indicating no significant difference. In the GEE sensitivity analysis, per-transfer biochemical pregnancy was nominally significant (aOR 1.876, 95% CI 1.028–3.424, P = 0.041) but did not survive Bonferroni correction (corrected P = 0.081); the clinical pregnancy rate was higher in the PGT-A group, but the difference was not statistically significant even before multiple-comparison correction (aOR 1.501, 95% CI 0.853–2.642, P = 0.158; Bonferroni-corrected P = 0.317).

#### For patients with >2 miscarriages

In the ITT population, the conservative cumulative live birth rate in this subgroup was 43.4% (75/173) in the PGT-A group and 49.0% (24/49) in the control group, with no statistically significant difference (P = 0.484). The optimistic cumulative live birth rate was 48.0% (83/173) in the PGT-A group and 57.1% (28/49) in the control group, also without statistical significance (P = 0.257). Given the small control group (n=49) and limited propensity-score overlap in this stratum, no adjusted estimate is presented.

In the PP population, for first-transfer outcomes, no significant differences were observed between the two groups in biochemical pregnancy rate (65.8% vs. 74.3%, P = 0.347), clinical pregnancy rate (59.5% vs. 62.9%, P = 0.720), or live birth rate (56.8% vs. 60.0%, P = 0.735). The conservative cumulative live birth rate was 67.6% (75/111) in the PGT-A group and 68.6% (24/35) in the control group, with no statistically significant difference (P = 0.912). Given the small sample size in the control group (n=35), this subgroup is reported descriptively only, and no multivariable adjustment, GEE sensitivity analysis, or Bonferroni correction was performed.

### Perinatal outcomes

Perinatal outcomes were assessed in the PP population among 166 deliveries (169 live-born neonates) in the PGT-A group and 117 deliveries (121 live-born neonates) in the IVF/ICSI group (**Table 8**); obstetric outcomes are reported per delivery, and birth weight and Apgar score per live-born neonate. No significant differences were observed between the two groups in obstetric complications, including gestational hypertension (8.43% vs. 8.55%; P = 0.973), gestational diabetes mellitus (7.23% vs. 6.84%; P = 0.899), premature rupture of membranes (3.61% vs. 7.69%; P = 0.132), or placenta previa (0.60% vs. 0.00%; P = 1.000). Cesarean delivery rates were also comparable (77.11% vs. 71.79%; P = 0.310). Preterm delivery before 37 weeks occurred in 14.46% and 15.38% of cases, respectively (P = 0.829), with very preterm delivery (28– < 32 weeks) rates of 1.20% and 3.42% (P = 0.393). Birth weight distributions were similar, with low birth weight (<2,500 g) observed in 9.47% vs. 9.92% (P = 0.898) and very low birth weight (<1,500 g) in 1.18% vs. 2.48% (P = 0.653). Monozygotic twin gestations accounted for 1.81% (3/166) and 3.42% (4/117) of deliveries, respectively (P = 0.638). Apgar scores at 1 minute were identical between groups (median 10, interquartile range 10–10; P = 0.310). No statistically significant between-group differences in perinatal outcomes were observed in the present sample.

**Table 8:** Perinatal outcomes of live births in the PGT-A and conventional IVF/ICSI groups in the per-protocol population.

Characteristics	PGT-A Group	IVF/ICSI Group	*P*-value
No. of deliveries (live birth) ^a^	166	117	
No. of live-born neonates ^b^	169	121	
Obstetric outcomes			
Gestational hypertension ^a^	8.43% (14/166)	8.55% (10/117)	0.973
Gestational diabetes ^a^	7.23% (12/166)	6.84% (8/117)	0.899
Hypothyroidism during pregnancy ^a^	0.60% (1/166)	0.00% (0/117)	1.000
PROM ^a^	3.61% (6/166)	7.69% (9/117)	0.132
Placenta previa ^a^	0.60% (1/166)	0.00% (0/117)	1.000
Cesarean delivery ^a^	77.11% (128/166)	71.79% (84/117)	0.310
Gestational age (weeks)			
Preterm delivery, <37 weeks ^a^	14.46% (24/166)	15.38% (18/117)	0.829
Very preterm delivery, ≥28 weeks and <32 weeks ^a^	1.20% (2/166)	3.42% (4/117)	0.393
Birth weight (g)			
Low birth weight, <2,500g ^b^	9.47% (16/169)	9.92% (12/121)	0.898
Very low birth weight, <1,500g ^b^	1.18% (2/169)	2.48% (3/121)	0.653
Monozygotic twin gestations (%) ^a^	1.81% (3/166)	3.42% (4/117)	0.638
Birth defects ^b^	0.59% (1/169)	0.00% (0/121)	1.000
1-minute Apgar Scores ^b^	10.00 (10.00, 10.00)	10.00 (10.00, 10.00)	0.310

PP, per-protocol; PGT-A, preimplantation genetic testing for aneuploidies; IVF/ICSI, *in vitro* fertilization/intracytoplasmic sperm injection; PROM, premature rupture of membranes.

Categorical variables are presented as n (%), and compared using the chi-square test or Fisher’s exact test where appropriate. Continuous variables are presented as median (interquartile range), and compared using the Mann–Whitney U test.

A total of 166 deliveries occurred in the PGT-A group, among which 3 were monozygotic twin gestations, yielding 169 live-born infants; in the IVF/ICSI group, 117 deliveries included 4 monozygotic twin gestations, yielding 121 live-born infants.

^a^Denominator is the number of deliveries (PGT-A, n = 166; IVF/ICSI, n = 117). ^b^Denominator is the number of live-born neonates (PGT-A, n = 169; IVF/ICSI, n = 121).

P < 0.05 was considered statistically significant.

## Discussion

### Principal findings

In this single-center retrospective cohort study, 589 women with uRPL undergoing their first oocyte retrieval with planned single blastocyst transfer were included, and PGT-A did not improve the CLBR per oocyte retrieval cycle. Under the ITT framework, the CLBR was 48.5% (166/342) in the PGT-A group versus 47.4% (117/247) in the conventional IVF/ICSI group (P = 0.779); after IPTW with doubly robust adjustment, the association remained non-significant (aOR 0.862, 95% CI 0.591–1.255, P = 0.437). Findings under the PP framework were consistent: neither live birth at first transfer (63.8% vs 58.3%, P = 0.272; aOR 0.942, 95% CI 0.570–1.556, P = 0.814) nor PP cumulative live birth (75.1% vs 69.6%, P = 0.230; aOR 0.858, 95% CI 0.487–1.512, P = 0.595) differed significantly between groups. In per-transfer sensitivity analyses using generalized estimating equations, no significant differences were found for biochemical pregnancy (aOR 1.112, 95% CI 0.685–1.806, P = 0.667), clinical pregnancy (aOR 1.055, 95% CI 0.670–1.661, P = 0.818), or live birth (aOR 0.980, 95% CI 0.630–1.525, P = 0.929). Neither age nor the number of previous miscarriages significantly modified the treatment association with CLBR in either framework (ITT: interaction P = 0.871 and P = 0.695, respectively; PP: P = 0.071 and P = 0.289; the miscarriage-number interactions are exploratory owing to limited propensity-score overlap in the >2-miscarriage stratum). The dominant feature of the PGT-A pathway was progressive attrition: 13.7% of retrieval cycles yielded no biopsy-suitable blastocyst, 21.0% of biopsied patients had no euploid embryo, and 54 patients consequently never reached embryo transfer. Among women aged ≥35 years and among those with exactly two previous miscarriages, unadjusted PP differences favored PGT-A; adjusted live birth and cumulative live birth were no longer significant. The nominally significant adjusted estimates for biochemical pregnancy and clinical pregnancy should be interpreted with caution after Bonferroni correction, and the significance observed for clinical pregnancy may reflect chance findings arising from multiple comparisons; among women who achieved a live birth, perinatal outcomes did not differ significantly between groups. Overall, in women with uRPL, PGT-A did not improve the chance of live birth per initiated cycle; the apparent per-transfer advantages observed in selected subgroups are more plausibly explained by embryo attrition and baseline imbalance than by a confirmed treatment benefit.

### Overall effectiveness of PGT-A in uRPL

The absence of cumulative benefit per oocyte retrieval is consistent with the largest randomized trial to date, which showed that PGT-A did not improve cumulative live birth rates compared with conventional IVF (8), and with retrospective and propensity score–matched studies in recurrent pregnancy loss populations reporting no improvement in CLBR per retrieval or per patient despite higher per-transfer rates (9, 10, 18, 37). Meta-analyses similarly indicate that any advantage of PGT-A is confined to per-transfer metrics, without a demonstrated cumulative benefit (4, 7). The present study extends this pattern to the uRPL population: because conventional single blastocyst transfer permits sequential use of all embryos obtained from a single retrieval, PGT-A merely reorders the sequence in which viable embryos are transferred rather than creating additional viable embryos; a per-transfer benefit is therefore inherently unlikely to translate into a per-retrieval benefit (37).

A second observation is that PGT-A could not be shown to reduce miscarriage rates: among first transfers in the PP population, the miscarriage rate per intrauterine clinical pregnancy was 4.7% (7/148) in the PGT-A group versus 5.8% (6/104) in the conventional IVF/ICSI group (P = 0.713). Had embryonic aneuploidy been the dominant cause of miscarriage in uRPL, euploid selection should have produced a clear reduction in miscarriage; the absence of such an effect is consistent with the etiological heterogeneity of uRPL and with evidence that euploidy rates do not decline with increasing numbers of previous miscarriages (13), although the higher blastocyst abnormality rates reported in young women with idiopathic RPL (12) indicate that aneuploidy is not entirely irrelevant in this setting. This interpretation is strictly hypothesis-generating.

### Interpretation of the ITT and PP frameworks

The divergence between the two frameworks is itself a central interpretive finding. The ITT analysis captures the complete pathway from oocyte retrieval onward, including attrition from all sources, whereas the PP analysis conditions on reaching embryo transfer—a post-treatment event influenced by PGT-A itself. Such conditioning can induce collider stratification; PP estimates, particularly the subgroup and interaction signals observed in PP, therefore cannot, in isolation, be interpreted as biological effect modification. In the PP first-transfer models, the age-by-treatment interaction was significant for biochemical pregnancy (P = 0.006) and clinical pregnancy (P = 0.004) and nominally significant for live birth (P = 0.040); however, the corresponding interaction was not significant for the primary outcome in either framework (ITT CLBR P = 0.871; PP CLBR P = 0.071). The interaction for number of miscarriages was likewise non-significant (ITT CLBR P = 0.695; PP CLBR P = 0.289); as prespecified, these miscarriage-strata interaction tests are exploratory, because near-violation of positivity in the >2-miscarriage stratum destabilizes the stratum-specific coefficients on which the tests are based. Therefore, we interpret the PP interactions as reflecting differential selection into transfer. The attrition underlying this selection was substantial. Of 342 PGT-A retrieval cycles, 47 (13.7%) yielded no biopsy-suitable blastocyst; among 295 patients who underwent biopsy, 62 (21.0%) had no euploid embryo, of whom 54 never underwent transfer. Approximately two-thirds of PGT-A patients ultimately reached a first transfer, compared with only about half of women aged ≥35 years (52/107 vs 65/111). Such dropout is documented in both randomized and observational studies (14, 15), and is predictable from baseline ovarian reserve and blastocyst yield (38); in uRPL, it directly offsets any per-transfer efficiency that embryo selection might confer.

At the time of data freeze, some patients still held untransferred cryopreserved embryos without having achieved a live birth: this applied to 4.7% (16/342) of the ITT population in the PGT-A group and to 6.8% (16/237) of those who had undergone at least one embryo transfer, versus 12.6% (31/247) and 15.6% (31/199), respectively, in the IVF/ICSI group. Importantly, such window-censored patients accounted for only a minority of all non-live-birth outcomes: of the 176 women in the PGT-A group and 130 women in the IVF/ICSI group who had not achieved a live birth by the follow-up cutoff, 160 (90.9%) and 99 (76.2%), respectively, had exhausted all embryos from the index retrieval or declined further transfer, and only 16 and 31 remained censored while still holding embryos. The ITT analysis counts these patients—censored by the limited observation window—as not having achieved a live birth, whereas the PP analysis excludes them. Because window censoring was more than twice as frequent in the IVF/ICSI group, classifying all such patients as having no live birth may disproportionately disadvantage the IVF/ICSI arm, thereby biasing the effect estimate toward PGT-A. Even so, despite this potentially favorable bias, the ITT analysis showed no superiority of PGT-A over IVF/ICSI, and the adjusted point estimate did not favor PGT-A (aOR 0.862, 95% CI 0.591–1.255, P = 0.437), which in turn reinforces the robustness of the null finding. The ITT results are therefore best interpreted as cumulative live birth per oocyte retrieval within the defined observation window, rather than as the final cumulative live birth rate after all cryopreserved embryos have been used. The PP analysis, by excluding patients who had not yet completed transfer of all available embryos owing to insufficient follow-up, may be biased in the opposite direction—toward IVF/ICSI—and, being conditioned on entering and progressing through the transfer process, is only partially representative of all patients who initiated a retrieval cycle. PP findings should thus be regarded as complementary and exploratory, interpreted alongside the ITT results and the distribution of remaining cryopreserved embryos, rather than as the primary basis for judging clinical superiority. Consistent with this interpretation, under the extreme assumption most unfavorable to the null hypothesis—counting all window-censored patients with remaining cryopreserved embryos as future live births—the optimistic CLBR remained non-significantly different between groups (53.2% vs 59.9%; aOR 0.691, 95% CI 0.470–1.017, P = 0.061), with the point estimate favoring conventional IVF/ICSI.

### Age-stratified effects

In women younger than 35 years, results were consistently null under both frameworks: ITT CLBR was 55.3% (130/235) versus 61.8% (84/136) (P = 0.226; aOR 0.791, 95% CI 0.481–1.303, P = 0.357), and PP CLBR was 76.9% (130/169) versus 81.6% (84/103) (P = 0.366; aOR 0.531, 95% CI 0.249–1.132, P = 0.101), with no significant differences in any first-transfer or per-transfer outcome. This finding is biologically plausible—aneuploidy rates are lowest in oocytes from younger women (11), leaving little room for selection to add value—and is consistent with the STAR trial, which found no advantage of PGT-A in good-prognosis patients (14).

In women aged ≥35 years, the ITT and PP results must be interpreted together because they answer different questions. There was no benefit per oocyte retrieval: ITT CLBR was 33.6% (36/107) versus 29.7% (33/111) (P = 0.534; aOR 0.846, 95% CI 0.447–1.603, P = 0.608). Within the PP population, unadjusted outcomes favored PGT-A—first-transfer live birth 63.5% (33/52) versus 43.1% (28/65) (P = 0.028) and CLBR 69.2% (36/52) versus 50.8% (33/65) (P = 0.044)—but after adjustment, the differences in live birth and cumulative live birth lost statistical significance (first-transfer live birth: aOR 1.932, 95% CI 0.818–4.561, P = 0.133; CLBR: aOR 1.543, 95% CI 0.639–3.728, P = 0.334), and the nominally significant adjusted estimates for biochemical pregnancy (aOR 2.657, 95% CI 1.049–6.730, P = 0.039) did not survive Bonferroni correction (corrected P = 0.079). Although the clinical pregnancy rate remained statistically significant after adjustment and Bonferroni correction (corrected P = 0.047), this finding should be interpreted with caution, given the limited sample size of this stratum and the inconsistency with the results for live birth and cumulative live birth rates, suggesting that it may reflect chance findings arising from multiple comparisons. The nominally significant GEE estimates for per-transfer biochemical pregnancy (aOR 2.483, 95% CI 1.067–5.781, P = 0.035) and clinical pregnancy (aOR 2.439, 95% CI 1.071–5.557, P = 0.034) in this stratum did not survive Bonferroni correction (corrected P = 0.070 and 0.068, respectively), and live birth did not differ significantly (aOR 1.725, 95% CI 0.802–3.709, P = 0.162); these signals are therefore interpreted as exploratory. The unadjusted PP comparison largely describes who passed through the attrition filter: only about half of PGT-A patients in this stratum reached transfer, so the transferred PGT-A subgroup had already cleared morphological, biopsy, and euploidy screening, whereas three-fifths of women in the control group received unscreened blastocysts. The disappearance of the difference after adjustment further suggests baseline prognostic imbalance. These data cannot confirm the age-specific benefit suggested by per-transfer meta-analytic results (7) or by the 35–40-year stratum of the STAR trial (14); they propose, but do not test, the hypothesis that selected older uRPL patients might derive per-transfer efficiency from PGT-A. In the opposite direction, under the optimistic assumption that all window-censored patients with remaining cryopreserved embryos would eventually achieve a live birth, the point estimates favored conventional IVF/ICSI—nominally significant in women aged ≥35 years (aOR 0.520, 95% CI 0.280–0.966, P = 0.039; Bonferroni-corrected P = 0.077) and in two pre-treatment specifications of the sequential adjustment analysis (aOR 0.622, P = 0.025 and aOR 0.583, P = 0.018; **Supplementary Table 4**); these sequential-adjustment specifications are presented as sensitivity analyses and were not part of the prespecified Bonferroni families, so their nominal P values should be interpreted with caution.

### Number of previous miscarriages

For women with exactly two previous miscarriages, the ITT analysis was null (CLBR 53.8% [91/169] vs 47.0% [93/198], P = 0.189; aOR 0.930, 95% CI 0.595–1.455, P = 0.752). Under the PP framework, unadjusted outcomes again favored PGT-A (first-transfer live birth 70.9% [78/110] vs 57.9% [77/133], P = 0.036; CLBR 82.7% [91/110] vs 69.9% [93/133], P = 0.021), but the adjusted estimates were non-significant for both first-transfer live birth (aOR 1.302, 95% CI 0.706–2.402, P = 0.398) and CLBR (aOR 1.139, 95% CI 0.564–2.300, P = 0.716). In the GEE models for this stratum, only per-transfer biochemical pregnancy reached nominal significance (aOR 1.876, 95% CI 1.028–3.424, P = 0.041), which did not survive Bonferroni correction (corrected P = 0.081); clinical pregnancy (aOR 1.501, 95% CI 0.853–2.642, P = 0.158) and live birth (aOR 1.365, 95% CI 0.796–2.340, P = 0.258) were not significant.

For women with more than two previous miscarriages, we report descriptive data only: in the ITT population, crude CLBR was 43.4% (75/173) versus 49.0% (24/49) (P = 0.484); in the PP population, 67.6% (75/111) versus 68.6% (24/35) (P = 0.912); first-transfer live birth rates were 56.8% (63/111) versus 60.0% (21/35; P = 0.735). Two features preclude adjusted inference in this stratum. First, the control group was small (49 ITT and 35 PP controls). Second, the number of previous miscarriages approximated a treatment-determining variable—approximately 80% of patients in the conventional IVF/ICSI group had exactly two miscarriages—so propensity score overlap in the >2-miscarriage stratum was limited, with several standardized mean differences exceeding 0.1 even after weighting. With positivity nearly violated, weighted estimates are unstable regardless of subsequent adjustment; we therefore draw no conclusion of benefit or harm in this stratum. Biologically, there is also little reason to expect efficacy to vary by miscarriage count, as euploidy rates appear unrelated to the number of previous miscarriages (13).

### Mosaic embryo transfer

Eleven transfer cycles (3.7% of PGT-A transfers) involved low-level mosaic embryos (30%–45% mosaicism): in eight patients no euploid embryo was available, and in the remaining three the mosaic embryo was transferred in the third transfer cycle after two euploid embryo transfers had not resulted in a live birth. Seven clinical pregnancies (63.6%) and six live births (54.5%) resulted, with one miscarriage. Among the six live births, four underwent amniocentesis with karyotyping and chromosomal microarray, all of which were normal (46,XN, with no pathogenic copy number variants); gestational ages at delivery ranged from 36 to 40 weeks and birth weights from 3,050 to 3,760 g, with no low-birth-weight infants. Although limited to eleven cycles, these outcomes are consistent with a prospective single-center series of one hundred mosaic embryo transfers demonstrating that healthy pregnancies can be achieved, albeit with lower efficiency than euploid transfers (39), with a larger series supporting the prioritization of low-level mosaic embryos (25), and with prospective non-selection data indicating that low- and intermediate-level mosaic embryos have developmental potential comparable to euploid embryos (26). Our findings therefore align with PGDIS and ESHRE guidance, which support considering low-level mosaic embryos—after appropriate genetic counseling and with prenatal diagnosis—when no euploid embryo is available (27, 40). Given the very small sample, no conclusion regarding the equivalence of mosaic and euploid embryo transfer can be drawn from our data; continued outcome registration and systematic prenatal follow-up remain essential.

### Perinatal outcomes

Among the 283 deliveries (290 live-born neonates) in the PP population (166 deliveries and 169 neonates after PGT-A; 117 deliveries and 121 neonates after conventional IVF/ICSI), no perinatal outcome differed significantly between groups, including hypertensive disorders of pregnancy (8.43% vs 8.55%, P = 0.973), gestational diabetes mellitus (7.23% vs 6.84%, P = 0.899), preterm birth (14.46% vs 15.38%, P = 0.829), low birth weight (9.47% vs 9.92%, P = 0.898), very low birth weight (1.18% vs 2.48%, P = 0.653), cesarean delivery (77.11% vs 71.79%, P = 0.310), and 1-minute Apgar score (median 10 in both groups, P = 0.310). This pattern is directionally consistent with meta-analyses and matched cohort studies reporting no increase in adverse obstetric or neonatal outcomes after trophectoderm biopsy (18, 19, 41, 42). Three qualifications are required. First, the comparison is conditioned on achieving a live birth and is therefore subject to selection bias. Second, rare outcomes such as birth defects (one case in the PGT-A group) occurred too infrequently to permit meaningful testing. Third, the absence of a significant difference is not evidence of equivalence. We therefore state only that no significant differences were observed in this sample; the perinatal safety of PGT-A in uRPL cannot be regarded as established.

### Strengths and limitations

Methodological strengths include a patient-centered primary endpoint—CLBR per oocyte retrieval defined according to ICMART/WHO terminology and reporting standards for infertility research (28, 29); parallel ITT and PP analyses that render selection into transfer explicit; robust variance estimation with GEE for repeated transfers; and complete reporting of event counts and denominators. Among 1,060 chromosome involvement events, chromosomes 16 (8.8%), 22 (8.1%), and 21 (7.6%) were most frequently involved, consistent with the cytogenetic spectrum of miscarriage tissue in RPL (43).

Several limitations remain. First, treatment allocation was not randomized; despite doubly robust adjustment, residual confounding may persist and should be considered together with the propensity score density plots and the distribution of stabilized weights. Second, the >2-miscarriage stratum approached violation of the positivity assumption; because miscarriage count nearly determined treatment allocation, this stratum is reported descriptively only. Third, PP estimates are conditioned on reaching transfer and are vulnerable to collider stratification. Fourth, this retrospective cohort enrolled consecutively within a defined time window without a prospective sample-size calculation. Previous studies reported small between-group differences in live birth rates (approximately 4%) (8, 9); the present cohort therefore had less than 80% power to detect a difference of that magnitude. Accordingly, the primary results should be interpreted on the basis of effect sizes and confidence intervals rather than P values alone: the adjusted estimate (aOR 0.862, 95% CI 0.591–1.255) cannot exclude a small benefit or harm, although any undetected difference is unlikely to be large. Fifth, per-transfer-cycle analyses are subject to selective censoring, because patients entering subsequent transfer cycles had not achieved a live birth in the preceding cycle; per-cycle results should therefore be interpreted descriptively. Sixth, multiple subgroup and secondary comparisons increase the risk of chance findings. Seventh, window censoring due to limited follow-up may bias the ITT estimate toward PGT-A, whereas the PP analysis, by excluding patients who had not completed transfer of all available embryos, may bias the estimate toward IVF/ICSI; both analyses should therefore be interpreted cautiously alongside the remaining cryopreserved embryos and their respective analytic populations. Eighth, the perinatal analysis was conditioned on live birth and was underpowered for rare events. Finally, this was a single-center study using a single PGT-A assay pipeline, with sequencing performed on the Ion PGM and Illumina NextSeq550 platforms; generalizability to other settings remains uncertain.

### Clinical implications and future directions

For unselected women with uRPL, these data do not support the routine use of PGT-A to improve the chance of live birth per oocyte retrieval, a position consistent with the caution expressed in current guidelines (1, 44). Counseling should make the attrition explicit: approximately one in seven retrieval cycles yields no biopsy-suitable blastocyst, one in five biopsied patients has no euploid embryo, and only about half of women aged ≥35 years ultimately reach transfer. Where PGT-A is still being considered—for example, by patients who prioritize fewer transfers over per-retrieval yield—the decision should be framed as a trade-off between possible per-transfer efficiency and the absence of proven cumulative benefit (5, 6), recognizing that the evidence for fewer transfers derives mainly from non-RPL populations (5). The unadjusted subgroup signals in older women and in women with two previous miscarriages are hypotheses for prospective testing, not indications for selective application. When no euploid embryo is available, mosaic embryo transfer may be a reasonable alternative, but only within a framework of structured informed consent and registry follow-up (27, 40). Future research should prioritize adequately powered randomized trials or multicenter cohorts with CLBR per oocyte retrieval as the primary endpoint, complete embryo accounting, prespecified stratified analyses, and long-term follow-up of children born after mosaic and euploid transfers.

## Conclusions

In this single-center cohort of women with unexplained recurrent pregnancy loss, PGT-A did not improve the cumulative live birth rate per oocyte retrieval compared with conventional IVF/ICSI, and this finding was consistent across intention-to-treat and per-protocol frameworks. Unadjusted per-transfer signals favoring PGT-A were attenuated after adjustment; although some adjusted biochemical and clinical pregnancy estimates remained nominally significant, they generally did not survive Bonferroni correction and should be regarded as exploratory; they should not drive routine use of PGT-A in this population. Given the substantial embryo attrition inherent to the PGT-A pathway, its procedural burden, and the absence of demonstrated cumulative benefit, treatment decisions should be individualized through explicit counseling about these trade-offs. Adequately powered prospective studies with cumulative live birth per retrieval as the primary endpoint are needed to determine whether any uRPL subgroup derives genuine benefit from PGT-A.

## Data Availability

The original contributions presented in the study are included in the article/**Supplementary Material**, further inquiries can be directed to the corresponding author/s.

## Supplementary material

Supplementary Figure 1 ITT Propensity Score Density Plot.

Supplementary Figure 2 PP Propensity Score Density Plot.

Supplementary Table 1 Outcomes of mosaic embryo transfers in the PGT-A group.

Supplementary Table 2 Standardized mean differences of baseline characteristics before and after inverse probability of treatment weighting.

Supplementary Table 3 Descriptive analysis of stabilized weights.

Supplementary Table 4 Sequential adjustment table for the primary outcome in the ITT population.
