# Supplementary material for: Cumulative live birth following preimplantation genetic testing for aneuploidy versus conventional IVF/ICSI in unexplained recurrent pregnancy loss: a retrospective cohort study with intention-to-treat and per-protocol analyses
Source: Front Endocrinol (Lausanne). 2026 Sep 30;17:1910686. doi: 10.3389/fendo.2026.1910686 (PMC13627900; doi:10.3389/fendo.2026.1910686)
Supplement: Supplementary file 1 [file Table1.docx]

**Supplementary Table 1. Outcomes of mosaic embryo transfers in the PGT-A group**

| **Patient** | **Maternal age (years)** | **Paternal age (years)** | **Day of embryo transfer** | **Embryo grade** | **Embryo genetic information** | **Pregnancy outcome** | **Amniocentesis and CMA results** | **Gestational age at delivery (weeks)** | **Birth weight (g)** |
| --- | --- | --- | --- | --- | --- | --- | --- | --- | --- |
| Patient 1 | 26 | 25 | D6 | 4BB | 21.43 Mb deletion at 2p25.3–p24.1 of chromosome 2 (mosaic level 38%) | Live birth | Karyotype: 46,XN; no pathogenic abnormalities identified on CMA | 36 | 3,750 |
| Patient 2 | 39 | 50 | D6 | 4BC | Mosaic trisomy 8 (mosaic level 32%) | No pregnancy | — | — | — |
| Patient 3 | 34 | 38 | D5 | 4AA | Mosaic monosomy 19 (mosaic level 45%) | Live birth | Karyotype: 46,XN; no pathogenic abnormalities identified on CMA | 40 | 3,200 |
| Patient 4 | 34 | 34 | D5 | 4AA | Mosaic trisomy 19 (mosaic level 30%) | No pregnancy | — | — | — |
| Patient 5 | 31 | 33 | D6 | 4BB | Mosaic partial deletion of chromosome 12, 26.80 Mb (mosaic level 37%) | No pregnancy | — | — | — |
| Patient 6 | 28 | 30 | D5 | 4AB | Mosaic trisomy 22 (mosaic level 32%) | Live birth | Not performed | 38 | 3,400 |
| Patient 7 | 29 | 29 | D5 | 4BB | 24.77 Mb deletion spanning 2p25.3–p23.3 of chromosome 2 (mosaic level 37%) | Miscarriage | — | — | — |
| Patient 8 | 31 | 32 | D5 | 4BB | Mosaic deletion of 70.80 Mb at 2q24.1–q36.2 of chromosome 2 (mosaic level 39%) | Live birth | Karyotype: 46,XN; no pathogenic abnormalities identified on CMA | 38 | 3,760 |
| Patient 9 | 25 | 29 | D6 | 3BB | Mosaic trisomy 6 (mosaic level 31%) | No pregnancy | — | — | — |
| Patient 10 | 25 | 27 | D5 | 4BA | Mosaic trisomy 22 (mosaic level 30%) | Live birth | Not performed | 39 | 3,300 |
| Patient 11 | 29 | 31 | D5 | 4BA | Mosaic trisomy 10 (mosaic level 30%) | Live birth | Karyotype: 46,XN; no pathogenic abnormalities identified on CMA | 39 | 3,050 |

CMA, chromosomal microarray analysis; D5/D6, day 5/day 6 of embryonic development. “—” indicates not applicable. All mosaic embryo transfers were single blastocyst transfers performed after genetic counseling.

Of the 11 patients, 8 had no euploid embryos available; in the remaining 3 patients, the mosaic embryo was transferred in the third transfer cycle after two prior euploid embryo transfers.

**Supplementary Table 2. Standardized mean differences of baseline characteristics before and after inverse probability of treatment weighting**

| **Characteristics** | **whole cohort (ITT)** | | **whole cohort (PP)** | |
| --- | --- | --- | --- | --- |
|  | **Pre-IPTW** | **After-IPTW** | **Pre-IPTW** | **After-IPTW** |
| Maternal age (years) | 0.24 | 0.05 | 0.29 | < 0.01 |
| Paternal age (years) | 0.24 | 0.04 | 0.32 | < 0.01 |
| BMI (kg/m^2^) | 0.13 | 0.13 | 0.23 | 0.21 |
| Duration of infertility(years) | 0.49 | 0.09 | 0.36 | 0.05 |
| Number of miscarriages | 0.53 | 0.04 | 0.51 | 0.05 |
| AFC | 0.37 | 0.05 | 0.42 | 0.03 |
| bFSH (IU/L) | 0.16 | 0.03 | 0.3 | 0.05 |
| bE2 (pmol/L) | 0.27 | 0.06 | 0.21 | 0.18 |
| bLH (mIU/mL) | 0.13 | 0.06 | 0.09 | 0.01 |
| **Infertility factor (%, n)** |  |  |  |  |
| Female factor | 0.61 | 0.06 | 0.6 | 0.11 |
| Male factor | 0.11 | 0.01 | 0.18 | 0.07 |
| Combined factors | 0.19 | 0.01 | 0.29 | 0.07 |
| Others | 0.28 | 0.05 | 0.17 | 0.02 |
| Total Gn dose (IU) |  |  | 0.43 | < 0.01 |
| Total Gn days (d) |  |  | 0.06 | 0.07 |
| Number of oocytes retrieved |  |  | 0.59 | 0.02 |
| IPTW, inverse probability of treatment weighting; ITT, intention-to-treat; SMD, standardized mean difference; BMI, body mass index; AFC, antral follicle count; bFSH, basal follicle-stimulating hormone; bE2, basal estradiol; bLH, basal luteinizing hormone; Gn, gonadotropin. | | | | |
| All values in the table represent standardized mean differences (SMDs) between the PGT-A group and the conventional IVF/ICSI group. An absolute SMD < 0.10 indicates good balance between groups, while SMD ≥ 0.10 suggests meaningful imbalance. | | | | |
| Continuous variables were standardized using Cohen's d; categorical variables were standardized using the formula for binary SMD. | | | | |

**Supplementary Table 3. Descriptive analysis of stabilized weights**

| **Characteristics** | **ITT PGT-A Pre-truncation** | **ITT PGT-A After-truncation** | **ITT IVF/ICSI Pre-truncation** | **ITT IVF/ICSI After-truncation** | **PP PGT-A Pre** | **PP PGT-A After** | **PP IVF/ICSI Pre** | **PP IVF/ICSI After** |
| --- | --- | --- | --- | --- | --- | --- | --- | --- |
| **Minimum** | 0.59 | 0.590 | 0.43 | 0.46 | 0.58 | 0.58 | 0.45 | 0.47 |
| **Maximum** | 6.51 | 3.640 | 62.41 | 3.64 | 5.78 | 4.31 | 6.97 | 4.31 |
| **Mean** | 0.99 | 0.980 | 1.20 | 0.96 | 1.00 | 1.00 | 0.98 | 0.96 |
| **Count (<1%)** | 0 | 0 | 5 | 0 | 0 | 0 | 3 | 0 |
| **Count (>99%)** | 1 | 0 | 4 | 0 | 1 | 0 | 2 | 0 |

**Supplementary Table 4. Sequential adjustment table for the primary outcome in the ITT population**

| **Characteristics** | **aOR (95% CI)** | ***P*-value** |
| --- | --- | --- |
| Conservative cumulative live birth rate^a^ | 1.048 (0.755~1.454) | 0.779 |
| Optimistic cumulative live birth rate^a^ | 0.761 (0.546~1.060) | 0.106 |
| Conservative cumulative live birth rate^b^ | 0.869 (0.598~1.265) | 0.464 |
| Optimistic cumulative live birth rate^b^ | 0.695 (0.473~1.021) | 0.064 |
| Conservative cumulative live birth rate^c^ | 0.862 (0.591~1.255) | 0.437 |
| Optimistic cumulative live birth rate^c^ | 0.691 (0.470~1.017) | 0.061 |
| Conservative cumulative live birth rate^d^ | 0.816 (0.549~1.212) | 0.312 |
| Optimistic cumulative live birth rate^d^ | 0.626 (0.413~0.948) | **0.027** |
| Conservative cumulative live birth rate^e^ | 0.788 (0.524~1.183) | 0.249 |
| Optimistic cumulative live birth rate^e^ | 0.584 (0.374~0.911) | **0.018** |
| Conservative cumulative live birth rate^f^ | 0.597 (0.391~0.911) | **0.017** |
| Optimistic cumulative live birth rate^f^ | 0.381 (0.236~0.615) | **<0.001** |
| ^a^No correction applied | | |
| ^b^Using only inverse probability weighting | | |
| ^c^Primary specification: inverse probability weighting with additional adjustment for BMI (kg/m2) | | |
| ^d^Adjusted for BMI (kg/m2), Maternal age (years) after inverse probability weighting | | |
| ^e^Adjusted for BMI (kg/m2), Maternal age (years), AFC and bFSH (IU/L) after inverse probability weighting | | |
| ^f^Adjusted for BMI (kg/m2), Maternal age (years), AFC, bFSH (IU/L) and Number of oocytes retrieved after inverse probability weighting | | |
| Note: specifications d,e and f adjust for covariates (maternal age, AFC, bFSH) that are already included in, or balanced by, the weighting model (post-IPTW SMD ≤ 0.05, Supplementary Table 2); they are presented for sensitivity display only and would not be entered under the doubly robust adjustment rule described in Statistical analysis. | | |
